# *Trypanosoma cruzi* Gene Expression in Response to Gamma Radiation

**DOI:** 10.1371/journal.pone.0029596

**Published:** 2012-01-11

**Authors:** Priscila Grynberg, Danielle Gomes Passos-Silva, Marina de Moraes Mourão, Roberto Hirata Jr, Andrea Mara Macedo, Carlos Renato Machado, Daniella Castanheira Bartholomeu, Glória Regina Franco

**Affiliations:** 1 Departamento de Bioquímica e Imunologia, Universidade Federal de Minas Gerais, Belo Horizonte, Minas Gerais, Brazil; 2 Departamento de Parasitologia, Universidade Federal de Minas Gerais, Belo Horizonte, Minas Gerais, Brazil; 3 Grupo de Genômica e Biologia Computacional, Centro de Pesquisas René Rachou, Fundação Oswaldo Cruz, Belo Horizonte, Minas Gerais, Brazil; 4 Instituto de Matemática e Estatística, Universidade de São Paulo, São Paulo, São Paulo, Brazil; 5 Instituto Nacional de Ciência e Tecnologia em Doenças Tropicais, Centro de Pesquisas René Rachou, Fundação Oswaldo Cruz, Belo Horizonte, Minas Gerais, Brazil; Biomedical Research Institute, United States of America

## Abstract

*Trypanosoma cruzi* is an organism highly resistant to ionizing radiation. Following a dose of 500 Gy of gamma radiation, the fragmented genomic DNA is gradually reconstructed and the pattern of chromosomal bands is restored in less than 48 hours. Cell growth arrests after irradiation but, while DNA is completely fragmented, RNA maintains its integrity. In this work we compared the transcriptional profiles of irradiated and non-irradiated epimastigotes at different time points after irradiation using microarray. In total, 273 genes were differentially expressed; from these, 160 were up-regulated and 113 down-regulated. We found that genes with predicted functions are the most prevalent in the down-regulated gene category. Translation and protein metabolic processes, as well as generation of precursor of metabolites and energy pathways were affected. In contrast, the up-regulated category was mainly composed of obsolete sequences (which included some genes of the kinetoplast DNA), genes coding for hypothetical proteins, and Retrotransposon Hot Spot genes. Finally, the tyrosyl-DNA phosphodiesterase 1, a gene involved in double-strand DNA break repair process, was up-regulated. Our study demonstrated the peculiar response to ionizing radiation, raising questions about how this organism changes its gene expression to manage such a harmful stress.

## Introduction

*Trypanosoma cruzi*, the etiologic agent of Chagas disease, is a kinetoplastid organism highly resistant to DNA damage caused by ionizing radiation [1], [2]. It is likely that this parasite has an efficient double-strand DNA break repair apparatus considering that after 500 Gy dose of gamma radiation the fragmented DNA is gradually repaired and the pattern of chromosomal bands is restored in less than 48 hours [2]. Remarkably, this dose is approximately 50–100 times higher than that tolerated by plant roots [3], *Plasmodium* [4], mammalian cells [5], and *Trypanosoma brucei*, the *T. cruzi* phylogenetic closest organism [6]. *Leishmania major*, another kinetoplastid, is similarly resistant to gamma rays since it is capable of growing by subculture after 300 Gy dose of gamma radiation, though slightly more sensitive when compared to *T. cruzi* [7]. A plausible hypothesis underlying this extraordinary recovery is the role played by the TcRAD51, a component of the homologous recombination machinery, which is induced following irradiation [2].

Time-course studies have been performed analyzing the overall gene expression in response to gamma rays in yeast [8], in *Arabidopsis thaliana* [9], in an Archaea organism [10], in human fibroblasts [11], and in *Deinococcus radiodurans*, an extremophile bacterium capable of enduring doses of gamma radiation higher than 15,000 Gy [12]. Moreover, little is known about global changes in gene expression during DNA repair and cell recovery mechanisms following ionizing irradiation in *T. cruzi*.

To address this question we used microarray experiments to explore global gene expression alterations in the first 96 hours after gamma radiation, when DNA repair has already been achieved. Amongst the gene expression patterns observed, we found an interesting prevalence in the up-regulated gene categories of members of the retrotransposon hot spot (RHS) gene family and kinetoplast mitochondrial DNA genes (kDNA). Remarkably, these genes increased their expression at 48 hours, when *T. cruzi* chromosomes are known to be fully reassembled [2]. In addition, translation and protein degradation processes are repressed, as well as other functional gene categories related to basal metabolism. These findings reveal, for the first time, how *T. cruzi* reacts to gamma radiation stress in terms of changes in its gene expression. Beyond its own characteristics, the radiation stress response presents several similarities to other types of stress responses, and this study may help to understand how this very peculiar parasite can handle hostile environments inside its vertebrate and invertebrate hosts.

## Methods

### *T. cruzi* epimastigote cell culture and gamma radiation

CL Brener strain epimastigote cells used in this study were isolated and characterized by Brener and Chiari [13] and have been maintained as frozen stocks at Universidade Federal de Minas Gerais. Cells were cultivated at 28°C in LIT medium (*Liver Infusion Tryptone* - *liver digest neutralized*) [14] supplemented with complement-inactivated 10% fetal bovine serum, streptomycin sulfate (0.2 g/L), and penicillin (200,000 units/L). Cultures with 5×10^8^ parasites in 20 mL of LIT medium (2×10^7^ cells/mL) were exposed to a dose of 500 Gy (1578 Gy/h per 20 minutes) in a cobalt (60Co) irradiator located at Centro de Desenvolvimento da Tecnologia Nuclear (CDTN), Belo Horizonte, Brazil. After irradiation, cells were counted in a cytometric chamber in specific time points to generate growth curves and further used for RNA extraction.

### RNA extraction, purification, and amplification

Epimastigote cells were subjected to RNA extraction using Trizol reagent (Invitrogen Life Technologies, USA) and RNA samples were purified using RNeasy® MiniEluteTM Cleanup Kit (Qiagen, Germany) according to the manufacturers' instructions. Total RNA was quantified using a Nanodrop ND-100 UV/Vis spectrophotometer (NanoDrop Technologies, USA) and the overall RNA quality was assessed by denaturing gel electrophoresis[15]. Total RNA (2 µg) was amplified using the Amino Allyl MessageAmp II kit (Ambion, USA), according to the manufacturer's specifications.

#### Experimental design

To evaluate the gamma radiation effect on *T. cruzi*, a triplicate of non-irradiated epimastigote cells (reference sample) and triplicates of irradiated cells (immediately after irradiation (i.a.i.), at 4, 24, 48, and 96 hours post-irradiation) had their total RNA extracted. Two independent biological experiments were performed (biological replicates). RNA extracted from triplicates in each time point was pooled before purification/amplification/labeling steps. The pool step was added to reduce biological variation and the cost by decreasing the number of microarray slides used. Each labeled RNA pool from the five time points after irradiation was hybridized against the reference RNA pool in each of the two biological experiments. Also, dye-swap technical replicates were performed for each time point. Thereby, four microarray slides for each time point were produced, two for each biological replicate and two for dye-swap replicates, totaling 20 slides.

The glass slide spotted with synthesized oligonucleotide DNA was kindly provided by Pathogen Functional Genomics Resource Center (PFGRC), a division of J. Craig Venter Institute, Rockville, MD. The arrays were coated with aminosilane. The arrays contained 12,288 oligonucleotides (70-mers), 500 *Arabidopsis thaliana* oligonucleotide (70-mers) controls spotted in duplicate, and 920 empty spots, totaling 26,496 spots. Considering the *T. cruzi* oligonucleotides, there were 10,616 annotated genes, including 5,791 genes encoding hypothetical or conserved hypothetical proteins and 1,672 obsolete sequences.

### aRNA labeling

Aminoallyl amplified RNA was labeled with Cy3 and Cy5 according to a modified version of the AminoAllyl MessageAmp II Kit (Ambion, USA) and TIGR's standard operational procedure – SOP #M008 (ftp://ftp.jcvi.org/pub/data/PFGRC/MAIN/pdf_files/protocols/M008.pdf). Briefly, we followed the manufacturer's instructions for the labeling step, but the initial amount of amplified RNA was changed to 8 µg. The Cy3 and Cy5 labeled samples were then combined. Labeled RNA was purified away from unincorporated dyes using YM-30 Microcon columns following manufacturer's specifications (Millipore®, USA). The final sample was dried again and resuspended in 30 µL of hybridization buffer (50% formamide, 5× SSC, 0.1% SDS, 0.1 M DTT, and 6% salmon sperm as blocking agent) according to TIGR's SOP #M008. The solution was heated to 95°C during 3 minutes and placed in ice for 30 seconds. After a brief centrifugation, the solution was dispensed onto the slide surface and covered with a coverslip.

### Slide hybridization and scanning

Slide pre-hybridization and hybridization steps were done as described elsewhere [16] with minor modifications. Briefly, slides were pre-hybridized by placing them in coupling jars containing pre-hybridization solution (5× SSC, 0.1% SDS, 1%BSA) at 42°C for one hour. Slides were washed twice by immersing 10 times in a beaker containing MilliQ water followed by dipping three times in isoamyl alcohol, and were subsequently spun dry. The slides containing 30 µL of samples in hybridization buffer were hybridized for 14 hours in a water bath at 42°C in the dark under cover slips inside Corning® hybridization chambers (Corning, USA). Slides were then washed two times for five minutes each in a low stringency wash solution (2× SSC, 0.1% N- Lauroylsarcosine) at 42°C (first wash) and RT (second wash), followed by two washes of five min in medium stringency wash (0.1× SSC, 0.1% N-lauroysarcosine) at RT and two washes for five minutes each in high stringency wash solution (0.1× SSC) at RT. Slides were spun dry and scanned using a microarray dual channel laser scanner (ScanExpress Lite da PerkinElmer®, USA) at 10 µm resolution, 100% laser power and PMT levels which were adjusted in order to obtain similar distributions of red and green signal intensities.

### Background correction, normalization, and statistical analysis

For each time point, gene expression analysis was done based on information obtained from four slides, one dye-swap pair for each biological replicate. ScanArray Express (PerkinElmer®) software was used to generate the slide images and raw intensity data that were then analyzed using specific packages from the R statistical language [17]. Spots of good quality (positive flag value) were considered to be analyzed. Data were inspected for spatial biases on both red and green channels (background and signal), for print-tip bias, dye bias, and bias dependent of intensity using the LIMMA [18] and marray [19] packages. Background correction was done with normexp method [20]. Robust spline and quantile methods were used for normalization within and between arrays, respectively. A linear model that incorporates biological and technical replication was used for statistical analysis. A list of differentially expressed genes was generated by applying adjusted p-values for multiple tests using ‘BH’ method, which works with the expected proportion of false-positives (FDR- False Discovery Rate) among the rejected hypothesis [18]. Graphics were drawn using GraphPad version 5.03. Heatmap and cluster graphics were created using gplots package and personal scripts (R statistical language). Microarray data generated were deposited in the Gene Expression Omnibus (GEO) database under the accession link http://www.ncbi.nlm.nih.gov/geo/query/acc.cgi?acc=GSE29510.

### GO functional analysis and enrichment

For the differentially expressed genes, functional assignment analysis for down- and up-regulated genes was performed using GOanna tool (http://agbase.msstate.edu/cgi-bin/tools/GOanna.cgi). GO terms returned by GOanna tool were used as inputs for GO-slim Viewer tool, generic subset. The GO-slim result was manually cured to remove redundancy (two or more equal gene products associated with the same GO-slim category) and to generate more specific categories when a category was quite generic.

### Obsolete sequence analysis

The *T. cruzi* microarray (version 2) slide was designed based on the first genome release of this species. Obsolete oligonucleotide sequences differentially expressed were aligned to the nuclear and kinetoplast genomes using blastn and were mapped into the annotated genome using Artemis software [21] in order to determine the localization.

#### Analysis of RHS genes

A two-tail Chi-square test was applied in each of the analyzed time-points to test whether *f*1 = *f*2 (null hypothesis) or *f*1≠*f*2 (alternative hypothesis) according to the equations 1 and 2 below:
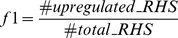
(1)

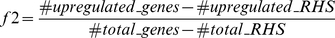
(2)where i) up-regulated RHS = RHS genes significantly up-regulated; ii) up-regulated genes = total number of significantly up-regulated genes; iii) total RHS = number of RHS probes spotted on the slide (379); and iv) total genes = number of gene probes spotted on the slide (12,288).

### qRT-PCR

A subset of differentially expressed genes was selected to be validated by qRT-PCR. Criteria used to selected genes were as follows: i) up or down regulated genes presenting at least 1.5 fold-change expression, ii) genes presenting significant differential gene expression (p<0.05) in at least one time point and iii) at least one representative of each gene cluster. We also included in qRT-PCR analysis some hypothetical genes and obsolete sequences according to special interests. Primers were designed using GenScript Real-time PCR (TaqMan) Primer Design (https://www.genscript.com/ssl-bin/app/primer) and Primer3 (http://frodo.wi.mit.edu/primer3/). Sequences are shown in Table S1. In order to test primer specificity, blastn was performed to align pairs of primers against NCBI nucleotide sequences database. The purified RNAs were treated with RQ1 RNase-Free DNase (Promega®, USA) and subsequently used for synthesis of cDNA using Superscript III cDNA Synthesis kit (Invitrogen™, USA), following the manufacturers' instructions. The qRT-PCR mix consisted of 10 ng of cDNA, 600 nM of each primer, 2.5 µL of Sybr Green PCR Master Mix (Applied Biosystems, Foster City, CA), and deionized H_2_0, totalizing 5 µL of reaction. Plates with 384 wells were read at 7900HT Fast Real Time PCR System (Applied Biosystems, USA). Primers for *T. cruzi* glyceraldehyde 3-phosphate dehydrogenase (GAPDH) were used as endogenous normalization control in all samples as reference applying the −2^ΔΔCt^ method [22]. Friedman and Dunn (post-test) tests were applied to verify the significance of the results. Spearman's rank coefficient was applied to verify the correlation between microarray and qRT-PCR results.

## Results

### Effect of gamma radiation in *T. cruzi* overall gene expression

Analysis of *T. cruzi* growth behavior in response to gamma-radiation revealed that, in both biological replicates, epimastigotes survived but did not grow during the whole time period analyzed (Fig. S1). However, despite the physical damage suffered by the cells (e.g. DNA fragmentation), they presented intense flagellar movement (visual observation) and remained viable.

**Figure 1:**
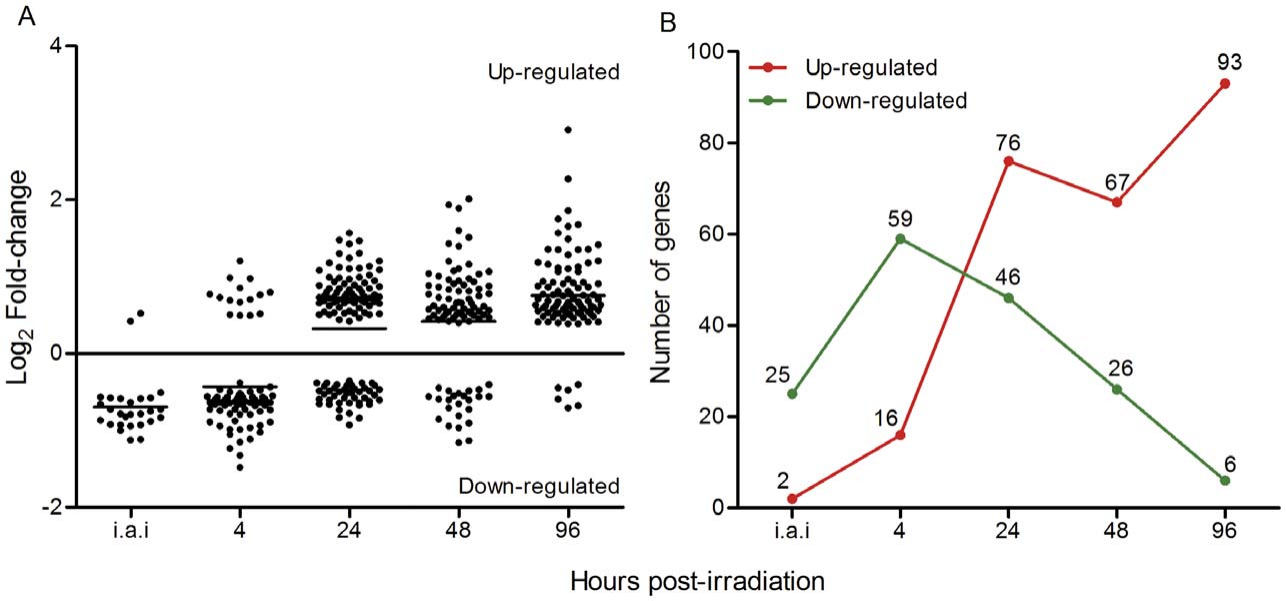
Number of differentially expressed genes in definite time points after *T. cruzi* irradiation. A) Scatter chart showing the distribution of fold-change values for statistically significant up-regulated genes over time points. B) Number of genes differentially expressed over time. ○: up-regulated, □: down-regulated. i.a.i: immediately after irradiation.

To visualize how gamma radiation affects *T. cruzi* overall gene expression we performed time-course microarray experiments in non-irradiated controls and in irradiated epimastigotes immediately after irradiation (i.a.i.), and at 4, 24, 48, and 96 hours after treatment. A dispersion graphic was generated using the fold-change values regarding the 273 genes found as being differentially expressed (adjusted p-value<0.05) in at least one time point. This result revealed a change in the expression pattern over time (Fig. 1A). At the first 4 hours, we observed a peak in the number of down-regulated genes (Fig. 1B).

It was also important to determine whether the overall decrease in gene expression in the first 4 hours after irradiation was due to a possible RNA degradation caused by gamma radiation that could compromise the labeling and hybridization steps. However, denaturing agarose gel electrophoresis revealed no difference on the rRNA (18S, 24Sα, and 24Sβ) band patterns (Fig. S2), demonstrating the high quality of total RNA in controls and irradiated samples (in triplicate) in both biological replicates.

**Figure 2:**
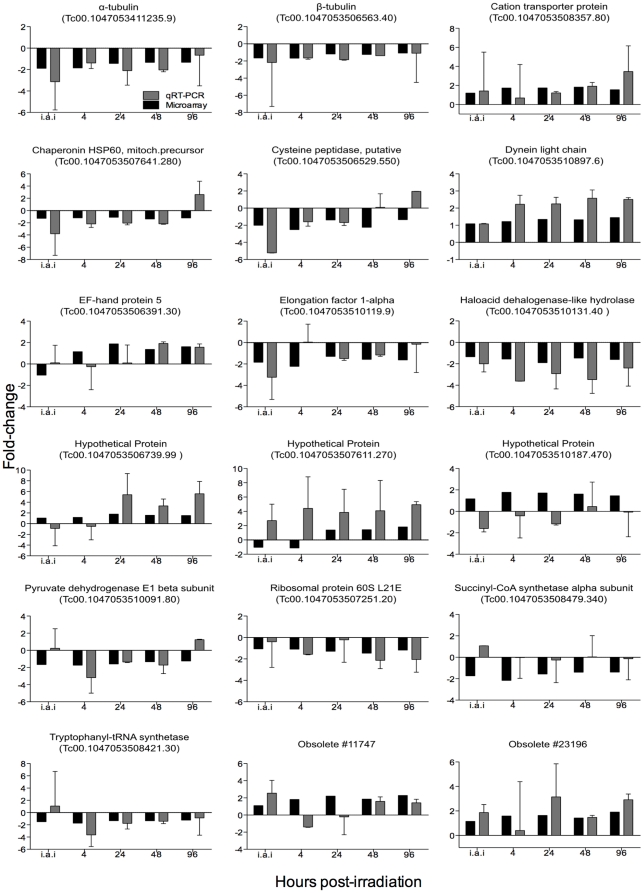
Comparisons of microarray and qRT-PCR fold-change values for selected genes. Black columns: microarray results. Gray columns: qRT-PCR. *: statistically significant results according to Linear model test for microarray and Friedman in association with Dunn tests for RT-PCR. i.a.i: immediately after irradiation.

Next, a subset of 18 differentially expressed genes was selected for validation using qRT-PCR (Fig. 2). Of those, 13 corresponded to known genes, three to hypothetical proteins, and two sequences were classified as obsolete. Obsolete sequences are those that, on a previous genome draft release, were initially considered to be derived from coding regions and therefore the corresponding oligos were spotted in the slide. However, after the release of the annotated *T. cruzi* CL Brener genome sequence [23], these oligonucleotides were, in fact, derived from non-coding regions [24]. Spearman's rank coefficient was applied to verify the correlation between microarray and qRT-PCR results. R^2^ ranged from 0.4298 to 0.7185 and all p-values were significant (Fig. S3). Primer sequences, slope, R^2^, and efficiency are shown in Table S1.

**Figure 3:**
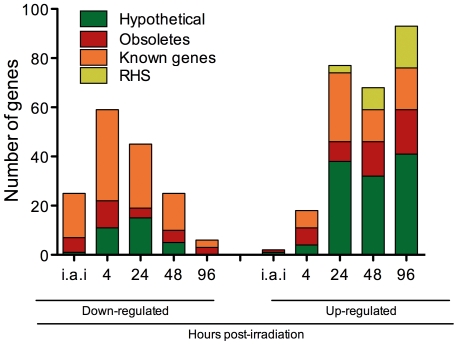
Categories of differentially expressed genes and their distributions along the time after *T. cruzi* irradiation. White: genes coding for hypothetical proteins. Light gray: obsolete sequences. Dark gray: genes coding for proteins with known function. Black: RHS genes. i.a.i: immediately after irradiation.

Genes down- or up-regulated coding for proteins with known function and/or predicted conserved domains corresponded to 41.4% of the total differentially expressed genes. Fold-change values for these genes are shown in Tables 1 and 2 respectively. RHS genes, a large family whose members are located preferentially at subtelomeric regions of *T. cruzi* chromosomes and present RIME/ingi insertion sites [25], corresponded to 7.7% of the total. The 50.9% remaining genes were found to be annotated as coding for hypothetical proteins, conserved or not (35.5%) or obsolete sequences (15.4%). Fig. 3 shows the distribution of these four categories for down- and up-regulated genes over time. Genes with predicted function (orange) were the most prevalent in the down-regulated gene category. In contrast, the up-regulated gene category was mainly composed of genes coding for hypothetical proteins (green), obsolete sequences (red), and RHSs (yellow). This last category was observed only at 24 hours post-irradiation.

**Table 1:** Fold-change of differentially down-regulated expressed genes coding for proteins with known function.

Gene ID	Product Name	Fold-change				
		i.a.i	4 h	24 h	48 h	96 h
Tc00.1047053509693.100	2- aminoethylphosphonate pyruvateamino-transferase-like protein	**−1.42**	**−1.51**	−1.14	−1.19	−1.24
Tc00.1047053507671.30	25 kDa translation elongation factor 1-beta	−1.61	**−1.55**	−1.09	−1.50	−1.34
Tc00.1047053504221.20	26S proteasome regulatory non-ATPase subunit	−1.05	−1.20	**−1.44**	−1.24	−1.10
Tc00.1047053504427.70	3-oxo-5-alpha-steroid 4-dehydrogenase	−1.10	−1.12	**−1.43**	−1.05	−1.06
Tc00.1047053506679.150	40S ribosomal protein S10	−1.04	−1.12	−1.16	**−1.36**	−1.17
Tc00.1047053510101.430	40S ribosomal protein S21	1.03	−1.10	−1.31	**−1.51**	−1.22
Tc00.1047053509353.30	40S ribosomal protein S3	1.00	−1.09	−1.17	**−1.47**	−1.20
Tc00.1047053506297.150	40S ribosomal protein S5	−1.06	−1.12	**−1.58**	−1.30	−1.23
Tc00.1047053510425.19	40S ribosomal protein SA	−1.04	−1.03	**−1.36**	**−1.27**	−1.23
Tc00.1047053503719.20	40S ribosomal protein SA	−1.03	−1.04	**−1.34**	−1.20	−1.05
Tc00.1047053505977.26	60S acidic ribosomal protein P2	−1.06	−1.12	**−1.30**	−1.27	−1.27
Tc00.1047053508461.490	60S ribosomal protein L23	1.06	−1.11	−1.35	**−1.46**	**−1.51**
Tc00.1047053506297.270	60S ribosomal protein L28	−1.06	−1.13	**−1.51**	−1.35	−1.23
Tc00.1047053511211.120	activated protein kinase C receptor	−1.16	−1.17	−1.32	**−1.32**	−1.11
Tc00.1047053411235.9	alpha tubulin	**−1.89**	**−1.85**	−1.43	−1.33	−1.32
Tc00.1047053510655.120	aminopeptidase P	−1.20	**−1.55**	1.17	−1.29	−1.02
Tc00.1047053506563.40	beta tubulin	**−1.65**	**−1.68**	−1.19	−1.24	−1.07
Tc00.1047053504153.160	carboxypeptidase	−1.50	**−1.58**	−1.19	−1.29	−1.02
Tc00.1047053507873.20	cell differentiation protein	−1.14	−1.19	**−1.30**	−1.13	−1.16
Tc00.1047053507641.280	chaperonin HSP60, mitochondrial precursor	**−1.92**	**−1.85**	−1.18	−1.40	−1.23
Tc00.1047053510187.420	chaperonin HSP60, mitochondrial precursor (pseudo)	−1.27	−1.19	−1.10	**−1.38**	−1.20
Tc00.1047053506247.50	chaperonin	−1.28	**−1.45**	−1.11	−1.17	−1.15
Tc00.1047053510187.270	COP-coated vesicle membrane protein erv25 precursor	−1.10	**−1.48**	−1.07	−1.09	−1.11
Tc00.1047053506529.550	cysteine peptidase	**−2.00**	**−2.50**	−1.38	**−2.23**	−1.35
Tc00.1047053508317.10	cysteine proteinase	−1.35	**−1.58**	−1.06	−1.13	−1.26
Tc00.1047053511391.160	cytochrome c1, heme protein, mitochondrial precursor	−1.42	−1.33	−1.13	**−1.63**	−1.39
Tc00.1047053510099.120	D-isomer specific 2-hydroxyacid dehydrogenase-protein	−1.07	−1.22	**−1.37**	−1.30	−1.21
Tc00.1047053510119.9	elongation factor 1-alpha (EF-1-alpha)	**−1.84**	**−2.22**	−1.30	**−1.57**	**−1.63**
Tc00.1047053508153.730	elongation initiation factor 2 alpha subunit	1.03	**−1.38**	−1.13	−1.06	−1.23
Tc00.1047053463269.10	eukaryotic translation initiation factor 1A	−1.17	−1.31	**−1.45**	−1.16	−1.07
Tc00.1047053506943.160	eukaryotic translation initiation factor 3 subunit 7-like protein	−1.06	−1.00	**−1.30**	1.05	1.01
Tc00.1047053506679.70	eukaryotic translation initiation factor 6 (eIF-6)	**−1.58**	**−1.92**	−1.33	−1.23	−1.14
Tc00.1047053511823.70	farnesyl pyrophosphate synthase	−1.43	**−1.55**	−1.32	−1.26	−1.21
Tc00.1047053511075.9	fatty acid desaturase	−1.41	**−1.59**	−1.11	−1.12	1.01
Tc00.1047053506661.30	fatty acid elongase	**−1.72**	**−1.65**	−1.40	−1.18	−1.30
Tc00.1047053507891.47	flagellar calcium-binding protein	−1.04	−1.02	−1.10	**−1.75**	−1.12
Tc00.1047053507547.90	glycosomal phosphoenolpyruvate carboxykinase	**−1.50**	**−1.98**	−1.18	**−1.43**	−1.06
Tc00.1047053508179.70	GPR1/FUN34/yaaH family	−1.31	−1.29	**−1.56**	−1.05	−1.01
Tc00.1047053503539.30	GTP-binding nuclear protein rtb2	**−2.18**	−1.37	1.16	−1.03	1.66
Tc00.1047053510131.40	haloacid dehalogenase-like hydrolase	−1.34	**−1.55**	**−1.90**	**−1.47**	**−1.60**
Tc00.1047053511041.40	hexose transporter	**−1.78**	**−1.98**	−1.02	−1.31	1.07
Tc00.1047053507943.40	histone H4	−1.11	**−1.35**	−1.25	−1.05	−1.09
Tc00.1047053509793.10	kinetoplast DNA-associated protein	−1.11	−1.19	**−1.46**	1.00	1.11
Tc00.1047053510667.14	membrane transporter protein	−1.09	−1.09	**−1.57**	1.03	−1.03
Tc00.1047053436521.9	mevalonate kinase	−1.19	−1.36	**−1.66**	−1.14	−1.19
Tc00.1047053436521.9	mevalonate kinase	−1.22	**−1.45**	−1.36	−1.18	−1.35
Tc00.1047053510155.20	mitochondrial RNA editing ligase 1	−1.18	**−1.48**	−1.22	−1.20	−1.19
Tc00.1047053508173.100	monooxygenase	−1.01	−1.16	**−1.40**	−1.22	−1.14
Tc00.1047053511817.40	NADH-cytochrome B5 reductase	**−1.90**	−1.49	1.21	1.06	−1.05
Tc00.1047053510645.20	nuclear transcription factor	−1.38	**−1.59**	−1.42	−1.14	−1.22
Tc00.1047053508173.180	nuclear transport factor 2 protein(NFT2)	−1.10	−1.31	**−1.52**	−1.27	−1.19
Tc00.1047053511573.58	nucleolar protein	−1.09	−1.17	**−1.78**	−1.07	−1.03
Tc00.1047053510859.17	nucleolar RNA-binding protein	−1.46	**−1.53**	−1.22	−1.17	−1.14
Tc00.1047053508707.200	nucleoside diphosphate kinase	−1.06	−1.02	−1.29	**−1.65**	−1.20
Tc00.1047053506773.50	nucleoside transporter-like	−1.33	**−1.52**	−1.09	−1.03	1.21
Tc00.1047053511355.30	phosphatidic acid phosphatase protein	−1.18	−1.21	**−1.79**	−1.02	−1.15
Tc00.1047053507617.9	prostaglandin F2alpha synthase	−1.22	**−1.66**	−1.08	−1.12	−1.01
Tc00.1047053508461.80	prostaglandin F2alpha synthase	**−1.72**	**−1.91**	−1.03	−1.32	−1.18
Tc00.1047053511635.40	protein tyrosine phosphatase	**−1.55**	**−1.67**	**−1.38**	−1.09	−1.12
Tc00.1047053510091.80	pyruvate dehydrogenase E1 beta subunit	**−1.65**	**−1.73**	**−1.59**	−1.34	−1.24
Tc00.1047053507251.20	ribosomal protein L21E (60S)	−1.06	−1.09	**−1.28**	**−1.46**	−1.18
Tc00.1047053508479.340	succinyl-CoA synthetase alpha subunit	**−1.73**	**−2.16**	**−1.56**	−1.39	−1.38
Tc00.1047053504045.60	thermostable carboxypeptidase 1	−1.26	**−1.52**	1.04	−1.08	−1.20
Tc00.1047053506855.260	thymidine kinase	−1.53	**−1.83**	−1.17	−1.35	−1.15
Tc00.1047053503555.30	trypanothione reductase	−1.33	**−1.39**	**−1.33**	−1.15	−1.16
Tc00.1047053508421.30	tryptophanyl-tRNA synthetase (pseudogene)	**−1.50**	**−1.73**	−1.33	−1.35	−1.24
Tc00.1047053503487.50	UDP-GlcNAc-dependent glycosyltransferase	1.12	−1.12	**−1.53**	1.05	−1.03
Tc00.1047053503929.10	V-type ATPase, A subunit	**−1.68**	**−1.58**	−1.22	−1.14	−1.14
Tc00.1047053511745.10	heat shock 70 kDa protein, mitochondrial precursor	1.08	1.02	−1.16	**−1.26**	−1.18
Tc00.1047053511715.100	pumilio/PUF RNA binding protein 7	1.06	1.04	**−1.71**	−1.06	1.02
Tc00.1047053509669.40	Zn-finger protein	−1.23	**−1.65**	1.11	−1.20	1.05

Bolded fold-changes: statistically significant values. i.a.i.: immediately after irradiation.

**Table 2:** Fold-change of differentially up-regulated expressed genes coding for proteins with known function.

Gene ID	Product Name	Fold-change				
		i.a.i	4 h	24 h	48 h	96 h
Tc00.1047053509941.100	2,4-dienoyl-coa reductase FADH1	1.10	1.29	**1.67**	1.33	1.32
Tc00.1047053504071.110	ama1 protein	1.01	1.17	1.41	1.44	**1.64**
Tc00.1047053508737.194	ARP2/3 complex subunit	1.03	1.24	**1.58**	1.40	1.33
Tc00.1047053508375.30	aspartate carbamoyltransferase	1.00	−1.13	**1.99**	−1.12	1.04
Tc00.1047053508183.4	aspartyl aminopeptidase	−1.09	1.09	**1.59**	1.05	−1.02
Tc00.1047053505997.70	ATPase	1.19	1.41	1.36	**1.59**	**1.50**
Tc00.1047053508903.100	ATPase	1.29	1.37	1.27	**1.52**	**1.88**
Tc00.1047053504149.20	ATP-binding cassette transporter ABCA1	**1.34**	1.24	1.06	1.06	1.07
Tc00.1047053506563.170	calpain-like cysteine peptidase (pseudo.)	−1.06	1.10	**1.38**	1.10	**1.31**
Tc00.1047053506563.210	calpain-like cysteine peptidase	1.03	1.19	1.43	**1.46**	1.23
Tc00.1047053508357.80	cation transporter protein	1.20	**1.74**	**1.75**	**1.82**	**1.55**
Tc00.1047053509455.140	cyclin	1.03	1.15	**1.67**	1.26	1.34
Tc00.1047053511421.110	developmentally regulated phosphoprotein	1.04	1.26	**1.59**	1.28	**1.59**
Tc00.1047053510687.10	dynein heavy chain (pseudogene)	1.07	−1.15	1.03	1.19	**1.47**
Tc00.1047053509585.10	dynein heavy chain	1.18	1.32	1.37	1.31	**1.56**
Tc00.1047053510897.6	dynein light chain lc6, flagellar outer arm	1.09	1.21	**1.34**	1.32	**1.45**
Tc00.1047053506391.30	EF-hand protein 5	−1.05	1.15	**1.88**	1.37	**1.61**
Tc00.1047053507483.20	EF-hand protein 5	−1.29	1.13	**2.15**	1.46	**1.56**
Tc00.1047053506529.508	glucose-6-phosphate isomerase, glycosomal	1.19	**1.66**	**2.69**	**2.06**	**1.92**
Tc00.1047053506529.508	glucose-6-phosphate isomerase, glycosomal	−1.03	1.34	**1.74**	**1.64**	1.32
Tc00.1047053508457.30	glycine cleavage system H protein	−1.11	1.15	**1.42**	1.15	1.33
Tc00.1047053510199.10	GP85-like protein	1.16	1.23	1.16	**1.32**	**1.54**
Tc00.1047053510659.240	lactoylglutathione lyase-like protein	1.04	1.35	1.57	**1.71**	1.36
Tc00.1047053510743.70	lactoylglutathione lyase-like protein	−1.23	1.32	**1.58**	1.32	1.11
Tc00.1047053473111.10	lathosterol oxidase	−1.11	1.65	**2.79**	1.98	**2.56**
Tc00.1047053508461.400	nucleoside diphosphate kinase	1.06	1.15	**1.67**	1.10	1.29
Tc00.1047053510105.100	UDP-glucose dehydrogenase	1.06	−1.02	**1.44**	1.16	1.17
Tc00.1047053508479.290	vacuolar sorting protein	1.07	−1.03	**1.89**	1.12	1.15
Tc00.1047053509069.30	tubulin binding cofactor A-like protein	−1.02	−1.02	1.52	1.78	**2.10**
Tc00.1047053506619.40	tyrosyl-DNA phosphodiesterase (Tdp1)	−1.00	1.01	**1.86**	1.29	1.32
Tc00.1047053503899.119	trypanothione/tryparedoxin dependent peroxidase 2	1.05	**1.33**	1.18	1.03	−1.06
Tc00.1047053508649.5	tryparedoxin peroxidase	−1.03	1.21	**1.46**	1.24	1.21
Tc00.1047053509907.60	trans-sialidase (pseudogene)	1.14	1.08	1.04	1.24	**1.41**
Tc00.1047053510121.130	serine/threonine protein kinase	1.41	1.07	1.29	**2.05**	1.77
Tc00.1047053511269.50	protein kinase A catalytic subunit	1.05	−1.11	**1.45**	1.34	**1.49**
Tc00.1047053508215.9	protein kinase C substrate protein, heavy chain	−1.09	1.14	**1.61**	1.39	1.24
Tc00.1047053504113.10	protein kinase	1.65	**1.62**	**1.60**	**1.42**	1.27
Tc00.1047053511671.80	protein kinase	1.29	1.38	1.12	**1.48**	1.41
Tc00.1047053507611.290	acetyltransferase	−1.04	−1.01	**1.42**	**1.40**	1.32
Tc00.1047053506357.50	alcohol dehydrogenase	−1.20	−1.17	**1.42**	1.16	1.11
Tc00.1047053507929.20	co-chaperone GrpE	1.09	1.16	**1.64**	−1.01	−1.03
Tc00.1047053506341.10	N-acetylglucosamine-6-phosphate deacetylase-like protein	1.23	**1.42**	1.03	−1.19	−1.17

Bolded fold-changes: statistically significant values. i.a.i.: immediately after irradiation.

### Gamma ray effect on the expression of obsolete sequences, genes of the maxi-circle kinetoplast DNA, and genes coding for RHS

**Figure 4:**
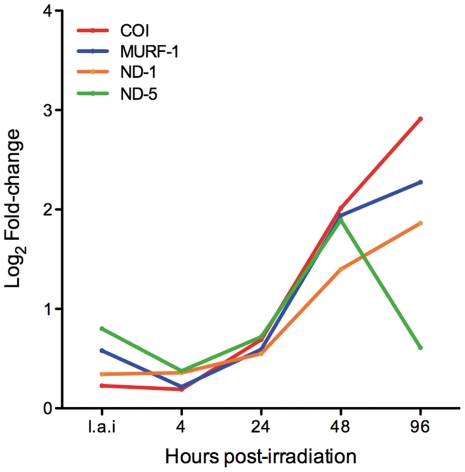
Time course expression of kDNA genes after *T. cruzi* cells irradiation. COI: Citochrome oxidase I; ND-1: NADH-dehydrogenase 1; ND-5: NADH-dehydrogenase 5; MURF-1 = Mitochondrial undefined reading frame 1. i.a.i: immediately after irradiation.

Of the 1,672 obsolete sequences spotted on the microarray slide, 42 (2.5%) were differentially expressed in at least one time point. This result prompted us to find out where in the genome these obsolete sequences were located. Thus, we used the Artemis software [21] to locate the oligonucleotides (70-mer) referring to obsolete sequences into the *T. cruzi* annotated genome (http://tritrypdb.org/common/downloads/release-2.5/Tcruzi/). We found that some sequences were located outside ORFs (seventeen sequences), some were located in contigs that could not be assigned to any particular chromosome (two sequences), some were found in at least two different genomic regions, e.g., multilocal sequences (nine sequences), and others were not located anywhere in the nuclear genome (14 sequences). Curiously, four sequences of this last category presented high values of fold-change (up to eight times), in particular at 48 and 96 hours post-irradiation (Fig. 4). A search against the non-redundant (nr) NCBI database using the blastx program identified these sequences as genes of the maxicircle kinetoplast mitochondrial genome (kDNA), which is composed of 18 genes [26]. The over-expressed kDNA gene code for three proteins of the mitochondrial respiratory chain (the subunits ND1 and ND5 of NADH dehydrogenase and the citochrome oxidase subunit one – COI), and for a mitochondrial unidentified reading frame (MURF-1). All four sequences presented a highly similar expression pattern, showing a substantial and significant increase in the transcript levels at 48 hours post-irradiation. The only exception was ND-5, which presented a decrease in gene expression 96 hours post-irradiation (Fig. 4). The remaining 14 kDNA genes were not represented in the microarray slide.

**Figure 5:**
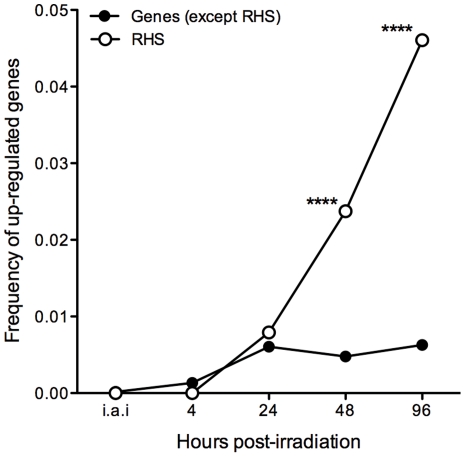
Frequency of RHS genes and non-RHS genes up-regulated in response to treatment with gamma rays over time. White squares: frequency of up-regulated RHS genes. Black circles: frequency of up-regulated genes, excluding RHS genes. i.a.i: immediately after irradiation. **** p<0.0001.

The *T. cruzi* genome project has identified 752 members of the RHS gene family, of which 557 are pseudogenes, and reported the presence of RHS orthologs in *T. brucei* [27], [28], *Trypanosoma congolense*, and *Trypanosoma vivax* [28]. We observed that 21 different RHS genes out of the 379 spotted on the microarray slide were up-regulated (Table S2): three at 24 hours, nine at 48 hours (one was the same as at 24 hours), and 17 at 96 hours post-irradiation (of which eight were expressed only at this time point). In contrast, RHS genes were not significantly down-regulated. Using the genome browser of the database TriTrypDB [28], we retrieved the genomic location of all 21 differentially expressed RHS genes. Ten are located on the edges of chromosomes, four in the central but repetitive chromosomal regions, and seven in contigs not embedded in the final genome assembly (Table S2). Two-sided Chi-square tests were independently performed for each time point and detected a significant association between RHS gene expression and the effect of gamma radiation (p<0.0001) at 48 and 96 hours (Fig. 5).

### Functional analysis of differentially expressed genes in epimatigote cells after irradiation

**Figure 6:**
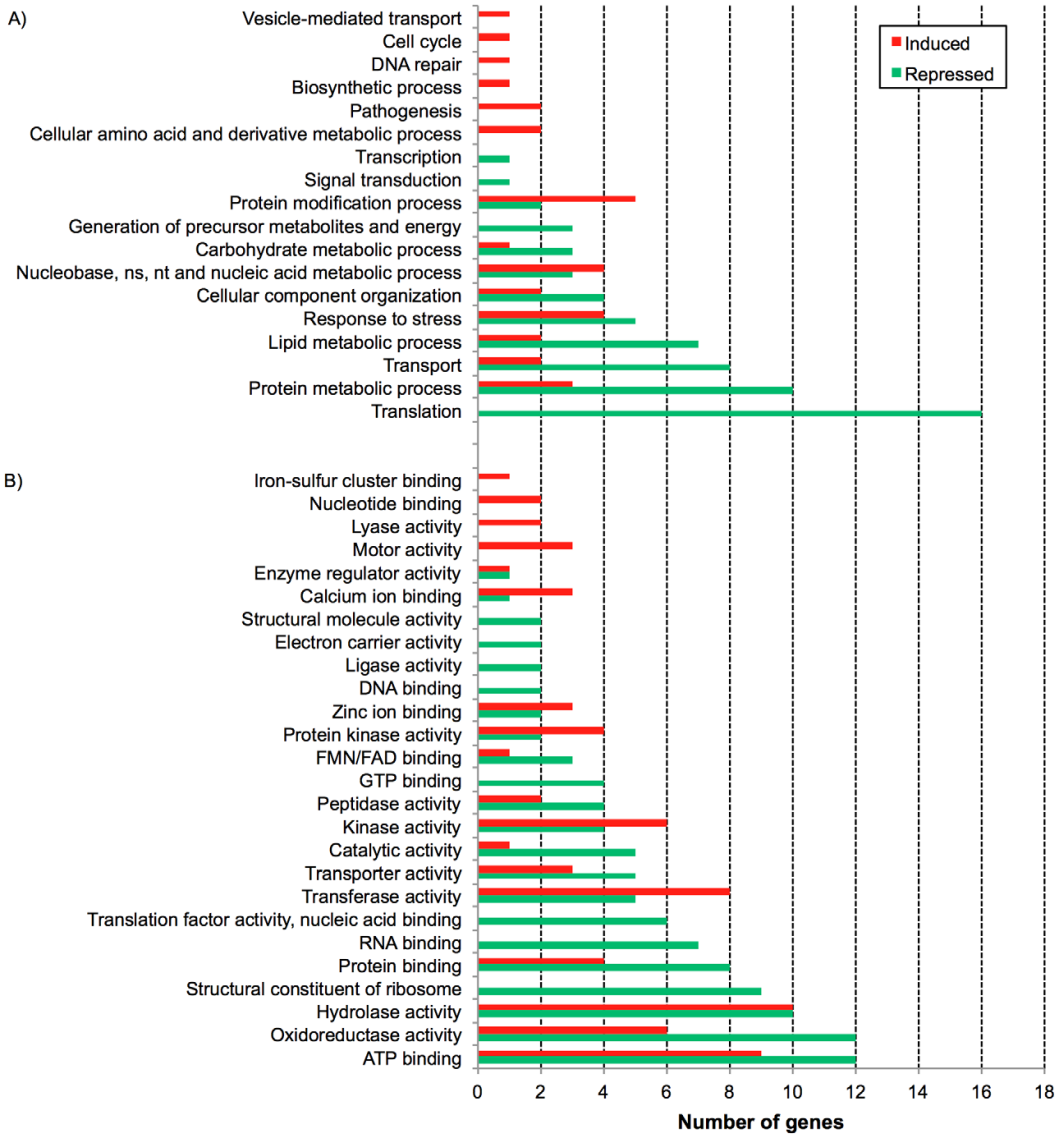
Gene Ontology Slim functional categories of differentially expressed genes. A) Biological Processes (BP) and B) Molecular Function (MF). Green bars: BP and MF categories repressed after gamma ray treatment. Red bars: BP and MF categories induced after gamma ray treatment. X axis: number of genes.

**Figure 7:**
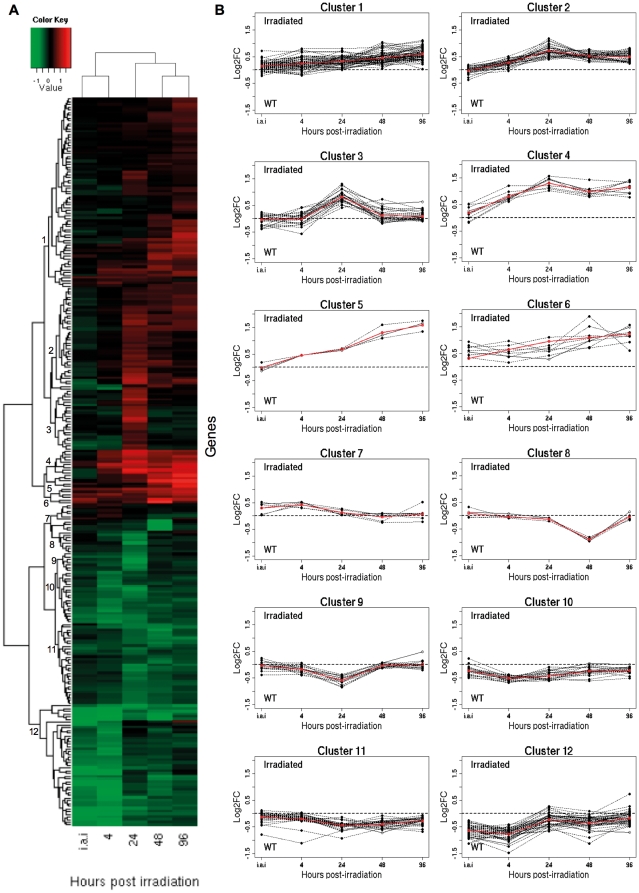
*T. cruzi* nuclear genes expression profiles. A) Heatmap of nuclear genes differentially expressed after *T. cruzi* irradiation. Shades of green indicate down-regulated and shades of red up-regulated genes. B) Clusters of genes based on similar expression profiles over time. Red lines: average fold-change values.

To identify the biological processes and molecular functions that were repressed or induced in response to ionizing radiation, Gene Ontology (GO) functional analysis was performed. GO-slim terms for biological processes (BP) and molecular function (MF) were assigned to differentially expressed genes and this procedure was essential to provide an overview of the effect of gamma rays in epimastigote cells (Fig. 6 and Table S3 for complete results). Concomitantly, a heatmap using all differentially expressed nuclear genes (269 genes) was generated to verify whether genes that participate in the same BP or MF showed similar gene expression profiles over time and could be grouped in the same cluster. Twelve different clusters were identified (Fig. 7). All clusters failed to group genes mapping at closer chromosomal locations (data not shown), and were constituted by genes from different BP or MF categories. An exception was seen in the cluster 11, which contains all genes from the MF category “structural constituent of ribosome”, along with genes from other MF and BP categories (Table S4).

According to GO functional analysis, the most prominent category affected was “protein translation,” which is clearly repressed given that the expression levels of seven translation initiation and elongation factors, as well as nine ribosomal constituents and a tryptophanyl-tRNA synthetase were significantly down-regulated in at least one time point (Fig. 6 and Table S3) and none were up-regulated. The two-sided Chi-square test indicated that the number of genes down-regulated associated with protein translation was not a random event (p = 0.0073).

The GO category “protein metabolic process” presented genes up- and down-regulated. Examples of down-regulated genes from this category were the heat shock proteins Hsp70 and Hsp60, chaperonin T-complex protein 1, aminopeptidase, carboxipeptidase, cysteine proteinase, cysteine peptidase, and a regulatory subunit of the 26S proteasome (Fig. 6 and Table S3). On the other hand, three genes were up-regulated (co-chaperone GrpE, aspartyl aminopeptidase, and calpain-like cysteine peptidase). The category “protein modification process” presented some up-regulated genes, mainly pertaining to the sub-categories “protein kinase activity” (e.g., serine/threonine protein kinase, protein kinase A, and other protein kinases) and “transferase activity” (e.g., acetyltransferase).

Some genes participating in pathways involved in “carbohydrate metabolic process” and in “generation of precursor of metabolites and energy” were down-regulated, such as a hexose transporter, pyruvate dehydrogenase E1 beta subunit, glycosomal phosphoenolpyruvate carboxykinase, one conserved hypothetical protein that seems to be a 2Fe-2S protein component of succinate dehydrogenase complex, succinyl-CoA synthetase, and V-type ATPase (Fig. 6 and Table S3). In general, transcripts coding for proteins involved in basic metabolic processes such as glucolysis, gluconeogenesis, acid citric cycle, ATP production, and lipid metabolism were reduced. In contrast, only one gene (glucose-6-phosphate isomerase) from the “carbohydrate metabolic process” category was over-expressed (Fig. 6).

## Discussion

An efficient DNA repair system may explain the *T. cruzi* impressive resistance to ionizing radiation, as it is able to reconstitute a completely fragmented karyotype in less than 48 hours after gamma radiation [2]. Our time course microarray analysis pointed to 273 statistically significant differentially expressed genes after parasite irradiation. These genes were classified into four different subsets: genes coding for proteins with known function (attributed to GO categories), genes for hypothetical proteins, genes for RHS, and obsolete sequences. More than 60% of the genes that are up-regulated code for proteins with unknown function. The remaining up-regulated genes comprised RHS and obsolete sequences. However, unlike a study conducted for *D. radiodurans*, where authors could infer function for uncharacterized genes based on operon context [12], analysis of the 12 *T. cruzi* gene clusters presenting similar expression profile demonstrated no correlation between chromosome location and pattern of gene expression over time. This indicates that *T. cruzi* genes are not transcribed as operon units. Indeed, no classical operons have been so far identified in this parasite, despite the fact that Trypanosomatid genes are transcribed as polycistronic units. These units may contain genes from unrelated pathways that, after trans-splicing processing, originate monocistronic mature transcripts with widely different expression levels [29].

Since kinetoplastids present very few transcription factors and no canonical RNA polymerase II promoter was identified in their genomes, these organisms control their gene expression at post-transcriptional and translational level [30]. Thus, it is likely that ionizing radiation does not considerably affect transcriptional rate in *T. cruzi*, which may explain, at least in part, the low fold-change values found for differentially expressed genes when comparing irradiated and non-irradiated cells (differentially expressed genes often showed less than 2-fold variation in transcript levels). Usually, such low values would be excluded from further analysis in other organisms. However, for parasites of Kinetoplastida order, previous microarray studies for *Trypanosoma* and *Leishmania* reported modest changes in the abundance of mRNA molecules, which were confirmed by qRT-PCR [31], [32]. They established that differentially expressed genes with low fold-change values, but with significant adjusted p-values for multiple tests are significantly differentially expressed. However, the effect of gamma radiation is very distinct in other eukaryotic organisms that control their gene expression mainly at transcription initiation. Accordingly, ionizing radiation causes an overall impact in the constituents of the transcription apparatus and transcription factors as reported for yeast [8] and *Arabidopsis thaliana* [9].

It is well known that Trypanosomatid mRNA's steady state is dictated primarily post-transcriptionally by mRNA stability, but the protein levels also depend on additional mechanisms to efficiently control translation initiation and elongation (translational selection) and protein turnover [29], [33], [34], [35]. Using a microarray transcriptomic analysis at different stages of differentiation to detected transcripts that were induced only during differentiation, Queiroz and collaborators have demonstrated that *T. brucei* genes form post-transcriptional regulons, in which mRNA encoding proteins playing roles in particular pathways, or encoding components of protein complexes, show a co-regulated expression [36]. Co-regulation of transcript levels may be achieved by binding of specific regulatory protein or RNA factors capable of controlling translation and mRNA decay [33], [34] to elements in the 3′-untranslated regions (3′-UTR). In this work, using the MEME suite software, we attempted to discover motifs at 3′-UTR of differentially expressed transcripts that could act as elements to control mRNA decay. Our results revealed no conserved motifs harbored by sequences from the same gene cluster (data not shown). This indicates that, if these clusters are composed of co-regulated genes (post-transcription regulons), their expressions are not coordinated by 3′-UTR elements.

Our functional analysis suggests that protein translation is reduced after irradiation and, thereafter, it is possible to speculate that the down-regulation of transcripts for the chaperone proteins hsp60 and hsp70 should be a consequence of the reduction in the rate of protein synthesis and processing. Such results are remarkably different from those reported in the literature, which showed that gamma radiation induces over-expression of heat shock proteins [37], [38], [39]. Also, an opposite effect in genes related to protein synthesis and protein fate was observed in the bacteria *D. radiodurans* after exposure to gamma radiation, in which DNA damage recovery is remarkably fast, with it being possible to observe genes up-regulated just 1 hour after treatment [40].

An increase in levels of transcripts for protein kinases, along with up-regulation of transcripts for proteins containing EF-hand motifs, a cation transporter protein, and a calpain-like cysteine peptidase (genes assigned as “protein metabolic and modification processes”) may suggest that *T. cruzi* is responding to radiation stress by activating signal transduction pathways involving calcium and protein phosphorylation. Indeed, our result regarding the calpain-like cystein peptidase agrees with a previous study that indicated a possible role of this gene in stress response in *T. cruzi*. It was strongly responsive to three different types of stress (nutritional, temperature, and acidic pH) and the protein presented no proteolytic activity, which is indicative of its involvement in signal transduction activity [41].

Previous work has shown that epimastigote growth halts after irradiation for at least 96 hours without cell division [2]. The arrest in cell growth may be explained by a decreased expression of α- and β-tubulin genes during all time points analyzed. These proteins assemble as the components of the cytoskeleton microtubules, which are involved in mitosis and cytokinesis. Similarly, depletion of cell division and motility has been described for other organisms after stress induced by ionizing radiation [9], [12]. Nevertheless, in spite of their growth arrest, epimastigotes were always active with an intense flagellar movement. A possible explanation may be related to the observation that genes associated with microtubule motor activity and components of the parasite swimming flagellum were up-regulated: the dynein light (lc6) and two dynein heavy chains [42], [43]. In fact, it was recently demonstrated that silencing of a putative inner arm dynein heavy chain resulted in flagellar immotility of *T. brucei* cells [44]. Noticeable is the fact that genes involved in lipid metabolism were also repressed, such as genes coding for enzymes involved in isoprenoid and sterol synthesis (mevalonate kinase, 3-oxo-5-alpha-steroid 4-dehydrogenase, and farnesyl pyrophosphate synthase), genes coding for enzymes required for the synthesis of long and unsaturated fatty acids (fatty acid desaturase, fatty acid elongase), and prostaglandin F2alpha synthase. Perhaps the growth arrest reflects a possible reduction in the levels of complex lipids as they are compounds of membranes, second messengers, or even cell mediators.

In order to recover from irradiation, *T. cruzi* needs to produce energy. However, some genes from “carbohydrate metabolic process” and “generation of precursor of metabolites and energy” were down-regulated. Perhaps *T. cruzi* basal metabolism is maintained at a minimum level after irradiation with a decrease in the transcript amount. Additionally, proteins involved in catabolic processes to generate energy might be saved from degradation. We also investigated nuclear genes coding for proteins from the respiratory chain but found that only cytochrome c1 was down-regulated. On the other hand, mitochondrial genes (ND1, ND5, and COI) may be over-expressed as consequence of a direct effect of the ionizing radiation in the mitochondrion, an organelle highly sensitive to oxidative stress [45]. Reinforcing this hypothesis is the finding that in the radioresistant human glioblastoma cell line T98G the levels of transcripts from cytochrome c oxidase subunits 1 and 2 and NADH dehydrogenase subunit 4 were raised after treatment with ionizing radiation, as consequence of oxidative stress [46].

Gamma radiation induces oxidative stress caused directly or indirectly via reactive oxygen species (ROS) production. ROS can also cause DNA single- and double-strand breaks and base damage. *T. cruzi* is naturally exposed to ROS produced by itself or by the host response to infection [47], and this parasite has a full system based on trypanothione-thiol metabolism to deal with oxidative stress[48]. It was observed that *E. coli* cells expressing the *T. cruzi* trypanothione showed an increased protection against genomic DNA damage (4.6-fold) and resistance to gamma rays (4.3-fold), as well as a decrease in intracellular levels of ROS [49]. Contrary to the expected finding, we found in this study that gamma radiation did not induce a massive expression of key antioxidant genes. Indeed, only two important genes (trypanothione/tryparedoxin dependent peroxidase 2 and tryparedoxin peroxidase) related to ROS removal were up-regulated. However trypanothione reductase, a key enzyme of the trypanothione-based thiol redox metabolism that reduces trypanothione disulfide back to its active state trypanothione, was down-regulated. We suppose that *T. cruzi* is experiencing oxidative stress and dealing with ROS species, although results did not indicate a robust stimulation in the expression of genes involved in oxidative damage repair.

It was reported that ionizing radiation induces the expression of TcRAD51 in *T. cruzi*. Moreover, epimastigotes overexpressing this gene halved the time necessary for full recovery after irradiation [2]. Interestingly, expression levels of most of the known *T. cruzi* DNA repair genes [23] were not noticeably affected (Table S5). Surprisingly, the tyrosyl-DNA phosphodiestarease I (tdr-1), a DNA repair enzyme that had not even been annotated as a DNA repair gene by the time the *T. cruzi* genome was published [23], is significantly up-regulated 24 hours post-irradiation, probably when the DNA repair process was already active. In the nucleus Tdr-1 hydrolyzes the linkage between the tyrosine of trapped topoisomerase 1 and the 3′ end of DNA, through the cleavage of 3′- phosphotyrosyl bonds [50]. This process, which is DNA ligase III alpha-dependent [51], prepares DNA double-strand breaks for DNA ligation and, consequently, DNA repair. Tdr-1 is also localized in the mitochondria, and the base excision repair (BER) mechanism necessary for an efficient oxidative damage repair depends on its action [52]. Recently, this enzyme was characterized in *Leishmania donovani*. The authors demonstrated that tdp-1 has a possible role in topoisomerase I-mediated DNA repair pathway both in the nucleus and in the kDNA. However no mechanism of action and possible interactive partners have been proposed so far for Trypanosomatids, considering that DNA ligase III is absent in kinetoplastids [53]. In any case, more studies will be needed to reveal the extent to which Tdp-1 participates in the cell recovery process: whether it acts in concert, in parallel, or independently of other genes.

Exposure to gamma rays is not naturally occurring during the different stages of the parasite life cycle. What is, thus, the reason why *T. cruzi* is so resistant to this kind of stress? Focusing on aspects of the *T. cruzi* life cycle, it is reasonable to imagine that the mechanisms underlying the parasite radiation resistance may be part of the responses against the stresses the organism faces such as its passage through the vector intestine, changes in temperature, pH and osmolarity, as well as insect saliva and blood digestion products [54]. Furthermore, blood digestion by the vector can provide heme molecule sub-products required for the Fenton's chemical reaction, a harmful free radical reaction that produces hydroxyl radical and causes oxidative stress. And last, just after a meal, the water present in the vector's blood is excreted by the Malphigian tubules in the intestine of the bug and the desiccated blood is stored in the crop [55]. Taking all together, the pathway though the vector's gut is a challenge for *T. cruzi* genome integrity.

One of the most intriguing questions of this study is how DNA is consistently reconstructed considering that chromosomal bands are extensively fragmented [2]. We showed that gamma radiation induces the overexpression of RHS genes/pseudogenes at 48 and 96 hours after irradiation. Coincidently, 48 hours is the time period when DNA repair is thought to be completed [2]. RHSs can be found preferentially in the repetitive subtelomeric region, but also in the center of chromosomes. However, other repetitive gene families such trans-sialidase-like family, MASP, gp63, and the putative surface protein DGF-1 (dispersed gene family-1) was not over-expressed as RHS. Our results suggest that the expression of members of this gene family may be related to the irradiation stress although it is not possible at this point to establish whether directly or indirectly. Similarly, in *S. cerevisiae*, genomic damage after exposure to gamma radiation induces Ty1 (LTR-retroelements) transcripts which reach to the impressive value of 86-fold after a dose of 800 Gy gamma rays when compared to non-irradiated cells. This increase in Ty1 transcripts activates retrotransposition in a dose-dependent manner [56]. Retrotransposition of Long Interspersed Elements (LINE-1s), mobile elements present in the human genome, is also increased after exposure to gamma rays and is responsible for genomic instability [57].

In conclusion, we showed that gamma rays affect the *T. cruzi* gene expression in a time-dependent manner. Expression of transcripts related to basal metabolic functions was reduced although apparently maintained at a minimum operation rate to meet the organism needs. Intriguingly, RHS, a category of proteins found preferentially in the repetitive subtelomeric region and in the center of chromosomes genes, were substantially induced although it is not clear whether or not they play a direct role in cell recovery and what role that would be. Further studies regarding the role of some of the genes found to be most potentially up- or down-regulated following irradiation, as well as genes known to be directly involved in DNA repair such as tdr-1 gene, will shed light on the repair/re-arrangement process after DNA fragmentation caused by the ionizing radiation.

## Supporting Information

Figure S1 ***T. cruzi*** **epimastigote cells (CL Brener strain) growth curve from two biological replicates.** Each time point corresponds to a median ± SD of a triplicate. •/▪ = Control cells (non-irradiated), ○/□ = Irradiated cells, ○/• = Biological replicate I, □/▪ = Biological replicate II. p.i = pre-irradiation. i.a.i = immediately after irradiation.
(TIF)

Figure S2 **Evaluation of** ***T. cruzi*** **RNA integrity after irradiation.** RNAs from biological replicates I and II (in triplicate, indicated as 1, 2, and 3) were submitted to formaldehyde-agarose gel electrophoresis and visualized after ethidium bromide staining. Control = non-irradiated cells, i.a.i = immediately after irradiation. In detail, sizes of the three rRNA bands.
(TIF)

Figure S3 **Spearman's correlation between microarray and qRT-PCR fold-changes.** Gray colored areas indicate the correlation between both techniques, where fold-change values are equally positive (upper gray area) or negative (lower gray area).
(TIF)

Table S1 **Sequences, slope, R^2^, and efficiency of primers used in qRT-PCR experiments.**
(DOC)

Table S2 **Fold-change of differentially expressed genes coding for proteins with unknown function, genes coding for RHS, and obsolete sequences.**
(DOC)

Table S3 **GO-Slim terms for Biological Processes (A and B) and Molecular Function (C and D) categories and their respective genes.**
(DOC)

Table S4 **Gene composition of each heatmap cluster.**
(DOC)

Table S5 **Fold-change values of DNA repair genes.**
(DOC)
