# Supplementary figures and images for: Trypanosoma cruzi Gene Expression in Response to Gamma Radiation
Source: PLoS One. 2012 Jan 11;7(1):e29596. doi: 10.1371/journal.pone.0029596 (PMC3256153; doi:10.1371/journal.pone.0029596)

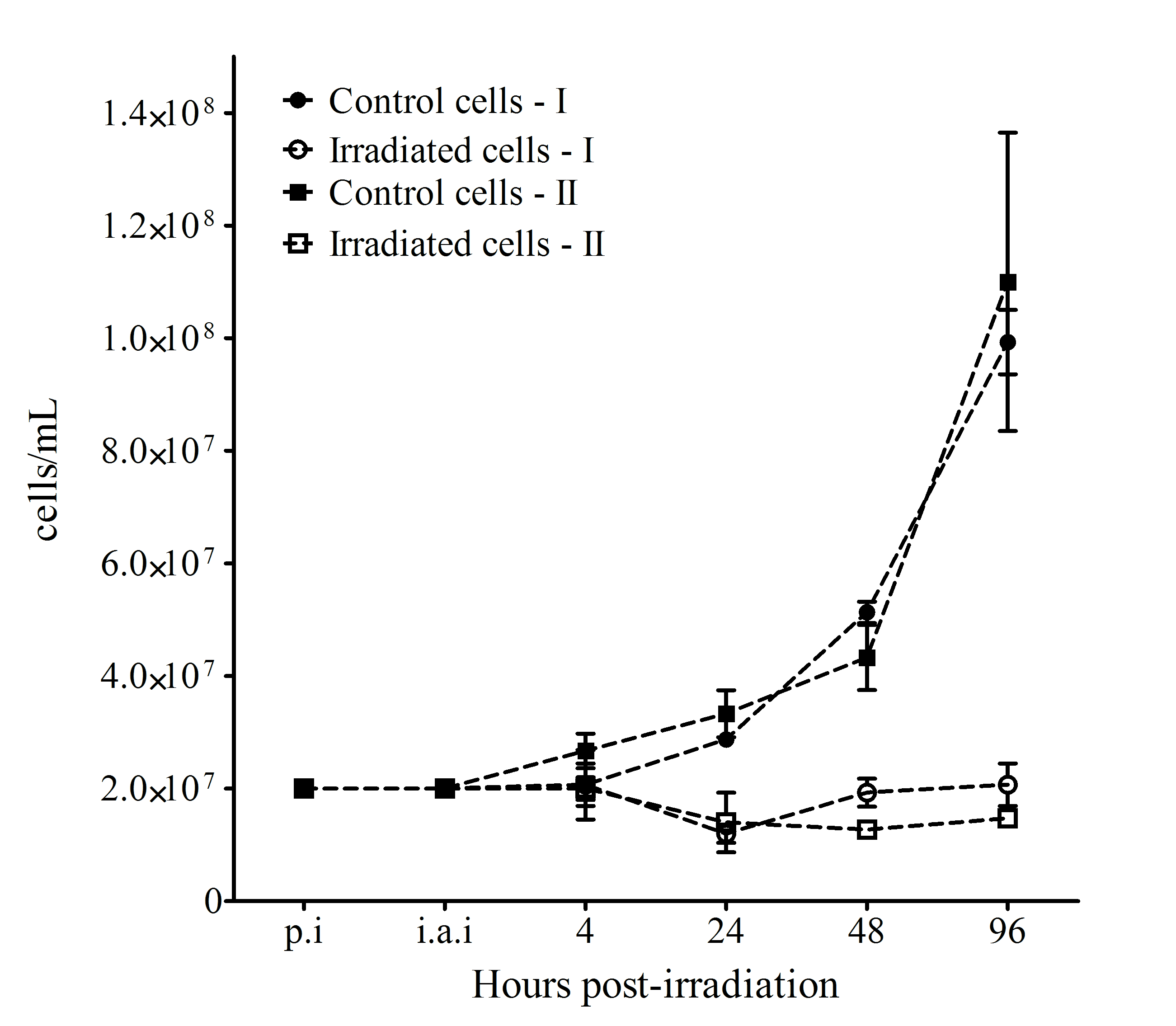

Supplement: Figure S1 — T. cruzi epimastigote cells (CL Brener strain) growth curve from two biological replicates. Each time point corresponds to a median ± SD of a triplicate. •/▪ = Control cells (non-irradiated), ○/□ = Irradiated cells, ○/• = Biological replicate I, □/▪ = Biological replicate II. p.i = pre-irradiation. i.a.i = immediately after irradiation. (TIF) [file pone.0029596.s001.tif]

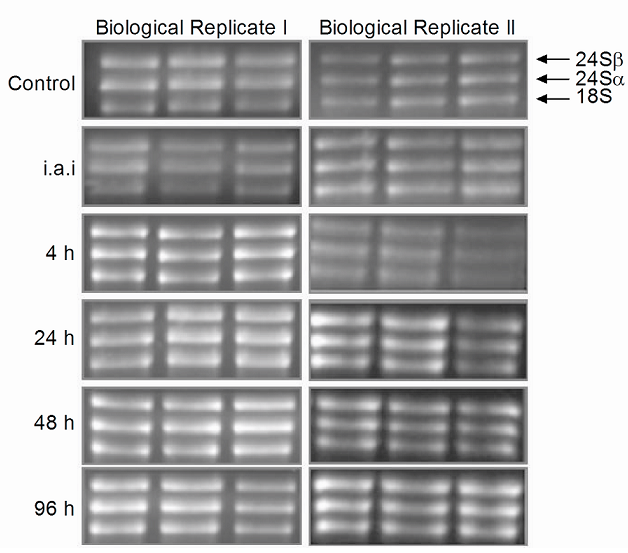

Supplement: Figure S2 — Evaluation of T. cruzi RNA integrity after irradiation. RNAs from biological replicates I and II (in triplicate, indicated as 1, 2, and 3) were submitted to formaldehyde-agarose gel electrophoresis and visualized after ethidium bromide staining. Control = non-irradiated cells, i.a.i = immediately after irradiation. In detail, sizes of the three rRNA bands. (TIF) [file pone.0029596.s002.tif]

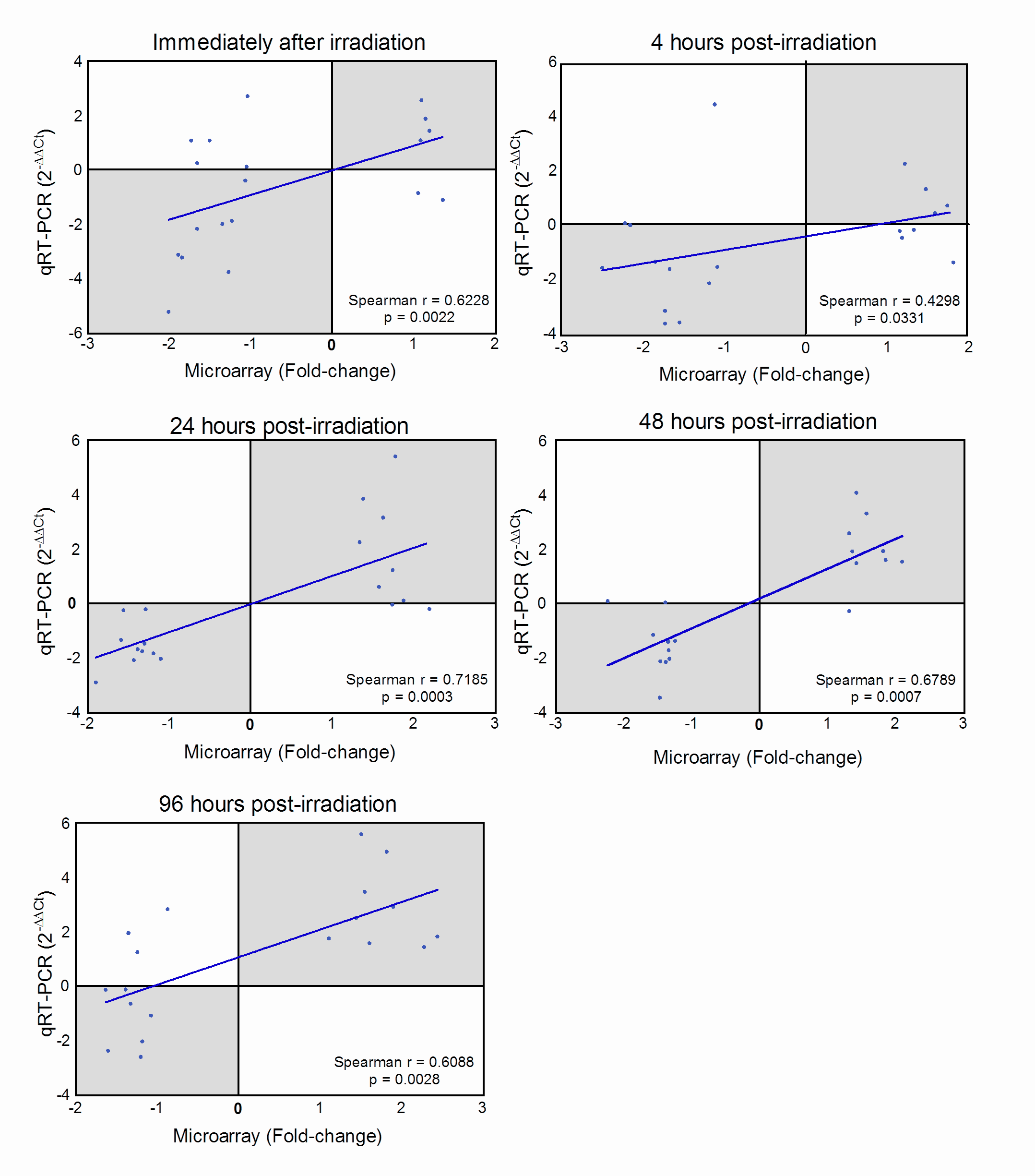

Supplement: Figure S3 — Spearman's correlation between microarray and qRT-PCR fold-changes. Gray colored areas indicate the correlation between both techniques, where fold-change values are equally positive (upper gray area) or negative (lower gray area). (TIF) [file pone.0029596.s003.tif]
