# Supplementary material for: Trypanosoma cruzi Gene Expression in Response to Gamma Radiation
Source: PLoS One. 2012 Jan 11;7(1):e29596. doi: 10.1371/journal.pone.0029596 (PMC3256153; doi:10.1371/journal.pone.0029596)
Supplement: Table S1 — Sequences, slope, R2, and efficiency of primers used in qRT-PCR experiments. (DOC) [file pone.0029596.s004.doc]

| Gene name | Gene ID | Primers sequences (5`-3`) | slope | R^2^ | Effic. |
| --- | --- | --- | --- | --- | --- |
| Alpha tubulin | Tc00.1047053411235.9 | F: GTTCTGTCTGGAGCACGGTA/R: CTCCGAGAAGAACGTGTTGA | -3.54 | 0.989 | 1.91 |
| Beta tubulin | Tc00.1047053506563.40 | F: AAAGAACATGATGCAGGCT/R: GTTCTGCACGTTGAGCATCT | -3.37 | 0.988 | 1.98 |
| Cation transporter protein | Tc00.1047053508357.80 | F:TGGGTGGACATCCATATGAC/R: AATGAGGCCATGCACAAATA | -4.32 | 0.942 | 1.70 |
| Chaperonin HSP60. mitoch. precursor | Tc00.1047053507641.280 | F: AGGTGTGCAACAAGACCAAC/R: CTTCATGTCAATTGGGTTCG | -3.92 | 0.996 | 1.78 |
| Cysteine peptidase | Tc00.1047053506529.550 | F: CGTGCGACGACACTAACTTT/R: GGGTAGCTTTCCTCCGTGTA | -3.26 | 0.984 | 2.02 |
| Dynein light chain lc6 | Tc00.1047053510897.6 | F: GCCAAAAGACATGGAGAAC/R: GGGGCCGTACTTCTTATCAA | -3.38 | 0.976 | 1.98 |
| EF-hand protein 5 | Tc00.1047053506391.30 | F: GATGCAAACGTGGATGGTAA/R :GTTGTAGTGGCATTGGCTTG | -3.41 | 0.969 | 1.96 |
| Elongation factor 1-alpha | Tc00.1047053510119.9 | F: GTCCTTGTCATTGCGTCATC/R: AGCACACAACCATCTGCTTC | -3.78 | 0.990 | 1.83 |
| Haloacid dehalogenase-like hydrolase | Tc00.1047053510131.40 | F: GAACGAGCATCACAGCATCT/R: ACATCGAAATAGGCACGAC | -3.29 | 0.979 | 2.01 |
| Hypothetical Protein | Tc00.1047053511201.40 | F: TCACAGCAACCTCAACAACA/R: CTCTTCAGGCAATCCCTTGT | -3.40 | 0.989 | 1.97 |
| Hypothetical Protein | Tc00.1047053507611.270 | F: CCGGACCAACTGGAAGTATT/R: CGGCGTGAAGTATCTCCATA | -3.62 | 0.981 | 1.89 |
| Hypothetical Protein | Tc00.1047053506739.99 | F: AGTTCTCTGAACGCCGTCTT/R: GTCTCTTTCCCGGTGTTGTT | -3.41 | 0.990 | 1.96 |
| Obsolete # 11747 | No gene ID | F: TTCACCGATTTCAGCAACTC/R: AGTACCCACGGGAATCTTTG | -4.00 | 0.983 | 1.75 |
| Obsolete # 23196 | No gene ID | F: CTATGGCCGGTTACTGTTCC/R: CCTGCCGTTTTCTTGTCTTT | -3.37 | 0.986 | 1.98 |
| Pyruvate dehydrogenase E1 beta subunit | Tc00.1047053510091.80 | F: GTCATGGAAAGTGAGGCATT/R: GCTACGGCAAGAACATCTGA | -3.20 | 0.989 | 2.05 |
| Ribosomal protein L21E (60S) | Tc00.1047053507251.20 | F: ATCTGTGAGCACCATCCTGA/ R: CAACACCACGAGGAGTAACG | -3.10 | 0.995 | 2.10 |
| Succinyl-CoA synthetase alpha subunit | Tc00.1047053508479.340 | F: ATCAAGAAGCCGGTGGTATC/ R: TGTACCCTGTCCACCAGAAA | -3.53 | 0.993 | 1.92 |
| Tryptophanyl-tRNA synthetase | Tc00.1047053508421.30 | F: ACTGCTGCACACAAAGTTCC /R: CCGTGTCGTGTAGTGTGATG | -3.39 | 0.981 | 1.97 |

**Ideally slope values should be between -3.1 and -3.6. R^2^ co-efficiency values should be as close as possible to 1. Effic. (efficiency of the reaction): E = 10^(-1/slope)^ –1. Values should be as close as possible to two-fold. The efficiency of the PCR should be between 90-110%. which means amplicon amount doubling at each cycle.**
