# Supplementary material for: Trypanosoma cruzi Gene Expression in Response to Gamma Radiation
Source: PLoS One. 2012 Jan 11;7(1):e29596. doi: 10.1371/journal.pone.0029596 (PMC3256153; doi:10.1371/journal.pone.0029596)
Supplement: Table S2 — Fold-change of differentially expressed genes coding for proteins with unknown function, genes coding for RHS, and obsolete sequences. (DOC) [file pone.0029596.s005.doc]

| Gene ID | Product Name | | Fold-change | | | | |  |
| --- | --- | --- | --- | --- | --- | --- | --- | --- |
|  |  |  | i.a.i | 4h | 24h | 48h | 96h | Complementary information |
| **Down-regulated** | | | | | | | | |
| Tc00.1047053447255.10 | | hypothetical protein. conserved | -1.36 | -1.17 | **-1.41** | -1.23 | -1.12 |  |
| Tc00.1047053503395.20 | | hypothetical protein. conserved | -1.19 | -1.11 | **1.65** | -1.03 | -1.02 |  |
| Tc00.1047053503687.30 | | hypothetical protein. conserved | -1.09 | **-1.41** | -1.15 | -1.23 | -1.06 |  |
| Tc00.1047053503703.30 | | hypothetical protein. conserved | -1.29 | **-1.60** | -1.29 | -1.23 | -1.09 |  |
| Tc00.1047053503809.120 | | hypothetical protein. conserved | -1.00 | -1.24 | **-1.52** | 1.02 | -1.11 |  |
| Tc00.1047053503897.120 | | hypothetical protein. conserved | -1.33 | **-1.50** | **-1.49** | -1.14 | -1.21 |  |
| Tc00.1047053504057.80 | | hypothetical protein. conserved | -1.22 | -1.09 | **-1.33** | -1.06 | -1.20 |  |
| Tc00.1047053506219.40 | | hypothetical protein. conserved | -1.43 | **-1.72** | -1.58 | -1.16 | -1.24 | Conserved domain: short chain dehydrogenase |
| Tc00.1047053506885.30 | | hypothetical protein. conserved | 1.25 | -1.07 | -1.15 | **-1.81** | 1.00 |  |
| Tc00.1047053506925.310 | | hypothetical protein. conserved | -1.17 | **-1.48** | **1.72** | 1.27 | 1.23 |  |
| Tc00.1047053507053.180 | | hypothetical protein. conserved | 1.18 | -1.29 | **-1.49** | **-1.47** | -1.24 |  |
| Tc00.1047053507221.30 | | hypothetical protein. conserved | -1.03 | -1.19 | **-1.40** | -1.26 | -1.10 |  |
| Tc00.1047053507509.40 | | hypothetical protein. conserved | **-2.17** | **-1.57** | -1.40 | -1.26 | -1.03 | Conserved domain: kinase protein |
| Tc00.1047053507747.94 | | hypothetical protein | -1.41 | **-1.43** | -1.19 | -1.22 | -1.01 |  |
| Tc00.1047053507993.380 | | hypothetical protein. conserved | -1.01 | -1.13 | **-1.40** | -1.25 | -1.15 | Conserved domain: signal recognition particle component |
| Tc00.1047053508173.264 | | hypothetical protein. conserved | -1.19 | **-1.30** | **-1.43** | -1.23 | -1.14 |  |
| Tc00.1047053508257.180 | | hypothetical protein. conserved | -1.03 | -1.06 | -1.06 | **-1.92** | -1.09 |  |
| Tc00.1047053508307.90 | | hypothetical protein. conserved | 1.18 | -1.12 | **-1.41** | -1.09 | -1.09 |  |
| Tc00.1047053508719.30 | | hypothetical protein. conserved | -1.07 | 1.01 | **-1.36** | -1.19 | -1.15 |  |
| Tc00.1047053509141.40 | | hypothetical protein. conserved | 1.10 | 1.05 | -1.06 | **-1.95** | 1.10 |  |
| Tc00.1047053509267.40 | | hypothetical protein. conserved | -1.14 | -1.38 | -1.45 | **-1.52** | -1.43 | Conserved domain: ARM motif |
| Tc00.1047053509769.60 | | hypothetical protein. conserved | -1.40 | **-1.54** | -1.46 | -1.24 | -1.12 | Conserved domain: 2F-2S protein. component of succinate dehydrogenase complex |
| Tc00.1047053510099.100 | | hypothetical protein. conserved | -1.12 | -1.16 | **-1.30** | -1.26 | -1.10 |  |
| Tc00.1047053510543.80 | | hypothetical protein | -1.04 | -1.05 | **-1.33** | -1.07 | 1.01 |  |
| Tc00.1047053510857.30 | | hypothetical protein. conserved | 1.13 | -1.32 | **-1.58** | -1.09 | 1.08 |  |
| Tc00.1047053511071.50 | | hypothetical protein. conserved (pseudo.) | -1.13 | **-1.41** | -1.28 | -1.33 | -1.20 | Conserved domain: HMG-CoA synthase |
| Tc00.1047053511517.120 | | hypothetical protein. conserved | -1.43 | **-1.56** | -1.31 | -1.27 | -1.32 |  |
| 03577 (Slide ID) | | Obsolete | -1.06 | -1.07 | **-1.41** | -1.24 | -1.09 | Repetitive |
| 04010 (Slide ID) | | Obsolete | -1.34 | **-1.47** | -1.05 | -1.13 | -1.12 | Not localized |
| 04685 (Slide ID) | | Obsolete | -1.10 | -1.13 | **-1.32** | **-1.40** | **-1.32** | Not localized |
| 05161 (Slide ID) | | Obsolete | -1.05 | -1.10 | **-1.56** | -1.19 | **-1.36** | Repetitive |
| 05692 (Slide ID) | | Obsolete | -1.28 | **-1.52** | -1.23 | -1.20 | -1.16 | Unstranslated region |
| 06316 (Slide ID) | | Obsolete | -1.46 | **-2.07** | -1.09 | -1.37 | -1.20 | Repetitive |
| 06794 (Slide ID) | | Obsolete | **-1.49** | **-2.03** | -1.18 | **-1.40** | -1.27 | Repetitive |
| 08330 (Slide ID) | | Obsolete | -1.06 | **-1.55** | -1.52 | -1.00 | -1.13 | Not localized |
| 09101 (Slide ID) | | Obsolete | -1.10 | -1.20 | -1.36 | **-1.52** | -1.33 | Not localized |
| 21242 (Slide ID) | | Obsolete | **-1.90** | **-2.79** | **-1.49** | **-1.87** | **-1.39** | Unstranslated region |
| 22884 (Slide ID) | | Obsolete | **-1.82** | **-2.35** | -1.41 | **-2.19** | -1.16 | Repetitive |
| 23089 (Slide ID) | | Obsolete | **-1.48** | **-1.46** | -1.23 | -1.13 | 1.01 | Repetitive |
| 23161 (Slide ID) | | Obsolete | -1.18 | **-1.48** | -1.18 | 1.03 | 1.00 | Not localized |
| 23282 (Slide ID) | | Obsolete | **-1.77** | **-1.95** | -1.03 | -1.12 | 1.13 | Not localized |
| 24338 (Slide ID) | | Obsolete | **-1.49** | **-1.65** | -1.06 | -1.11 | -1.04 | Not localized |
| **Up-regulated** | | | | | | | | |
| Tc00.1047053478463.10 | | hypothetical protein | 1.21 | 1.20 | 1.19 | **1.36** | **1.63** |  |
| Tc00.1047053503395.20 | hypothetical protein. conserved | | -1.19 | -1.11 | **1.65** | -1.03 | -1.02 |  |
| Tc00.1047053503415.40 | | hypothetical protein | -1.09 | 1.19 | **1.99** | **1.51** | 1.21 |  |
| Tc00.1047053503419.54 | hypothetical protein. conserved | | 1.12 | 1.03 | **1.67** | -1.04 | -1.06 |  |
| Tc00.1047053503839.19 | hypothetical protein. conserved | | 1.11 | 1.05 | **2.47** | 1.04 | 1.03 | Conserved domain: B-box zinc-finger conserved domain |
| Tc00.1047053503841.20 | hypothetical protein. conserved | | 1.08 | -1.04 | **1.83** | 1.01 | -1.02 | CBS conserved domain |
| Tc00.1047053503911.30 | | hypothetical protein. conserved | 1.14 | 1.43 | **2.12** | 1.46 | 1.45 |  |
| Tc00.1047053503925.6 | hypothetical protein. conserved | | -1.22 | 1.20 | **2.27** | 1.43 | **1.76** |  |
| Tc00.1047053503975.100 | hypothetical protein. conserved | | 1.11 | 1.17 | **1.66** | **1.37** | 1.21 |  |
| Tc00.1047053504005.54 | hypothetical protein. conserved | | -1.08 | 1.07 | **1.36** | **1.41** | **1.52** |  |
| Tc00.1047053504037.10 | hypothetical protein. conserved | | -1.13 | -1.02 | **1.67** | **1.64** | **1.55** |  |
| Tc00.1047053504227.10 | hypothetical protein. conserved | | 1.05 | 1.19 | **1.44** | -1.03 | -1.01 |  |
| Tc00.1047053504433.20 | | hypothetical protein. conserved | 1.42 | 1.53 | **2.77** | **1.76** | **2.17** |  |
| Tc00.1047053506133.179 | | hypothetical protein | 1.13 | 1.19 | 1.20 | **1.85** | **2.15** |  |
| Tc00.1047053506195.290 | | hypothetical protein. conserved | 1.03 | **1.41** | 1.20 | 1.03 | 1.07 |  |
| Tc00.1047053506713.24 | hypothetical protein. conserved | | 1.10 | 1.21 | 1.27 | 1.22 | **1.33** |  |
| Tc00.1047053506739.99 | hypothetical protein. conserved | | 1.06 | 1.18 | **1.78** | **1.58** | **1.51** |  |
| Tc00.1047053506773.104 | hypothetical protein. conserved | | -1.08 | 1.27 | 1.42 | **1.50** | **1.53** |  |
| Tc00.1047053506789.270 | hypothetical protein. conserved | | 1.18 | 1.03 | **1.98** | -1.10 | -1.04 |  |
| Tc00.1047053506825.200 | | hypothetical protein | 1.03 | 1.18 | 1.36 | **1.54** | 1.45 |  |
| Tc00.1047053506829.90 | | hypothetical protein. conserved | -1.13 | 1.59 | **2.97** | **2.70** | **2.55** |  |
| Tc00.1047053506831.63 | hypothetical protein. conserved | | -1.04 | 1.23 | **1.68** | 1.52 | 1.48 |  |
| Tc00.1047053506925.310 | hypothetical protein. conserved | | -1.17 | **-1.48** | **1.72** | 1.27 | 1.23 |  |
| Tc00.1047053506947.50 | hypothetical protein. conserved | | 1.25 | 1.16 | 1.06 | 1.19 | **1.46** | Conserved domain: major facilitator superfamily conserved domain |
| Tc00.1047053507053.140 | hypothetical protein. conserved | | 1.00 | -1.03 | **-1.38** | -1.09 | -1.07 |  |
| Tc00.1047053507083.10 | | hypothetical protein. conserved | 1.13 | **1.63** | **2.03** | **1.72** | **1.88** |  |
| Tc00.1047053507083.70 | hypothetical protein. conserved | | 1.03 | 1.13 | 1.14 | 1.04 | **1.64** |  |
| Tc00.1047053507159.30 | hypothetical protein. conserved | | 1.09 | 1.12 | **1.52** | 1.26 | 1.30 |  |
| Tc00.1047053507165.30 | | hypothetical protein. conserved | -1.03 | 1.46 | 1.42 | 1.67 | **1.84** | Conserved domain: EF-hand conserved domain |
| Tc00.1047053507275.24 | hypothetical protein. conserved | | 1.08 | 1.17 | 1.15 | 1.21 | **1.31** |  |
| Tc00.1047053507491.140 | hypothetical protein. conserved | | -1.06 | 1.28 | **1.85** | 1.27 | 1.24 |  |
| Tc00.1047053507521.60 | hypothetical protein. conserved | | 1.00 | 1.27 | 1.30 | 1.21 | **1.60** |  |
| Tc00.1047053507611.270 | hypothetical protein. conserved | | -1.03 | -1.12 | 1.39 | 1.42 | **1.82** |  |
| Tc00.1047053507875.30 | hypothetical protein. conserved | | -1.05 | -1.01 | **1.93** | -1.08 | -1.04 |  |
| Tc00.1047053508059.50 | hypothetical protein. conserved | | -1.04 | 1.04 | **1.54** | **1.68** | **1.61** |  |
| Tc00.1047053508061.10 | | hypothetical protein | 1.08 | 1.11 | 1.06 | 1.23 | **1.54** |  |
| Tc00.1047053508153.364 | hypothetical protein. conserved | | -1.06 | 1.19 | **1.80** | 1.25 | 1.29 |  |
| Tc00.1047053508293.130 | | hypothetical protein | 1.18 | 1.10 | 1.08 | **2.01** | **1.60** |  |
| Tc00.1047053508319.10 | | hypothetical protein | -1.05 | 1.37 | 1.60 | **2.15** | **2.55** |  |
| Tc00.1047053508479.330 | | hypothetical protein. conserved | -1.01 | 1.38 | **1.51** | **1.44** | **1.43** |  |
| Tc00.1047053508707.300 | hypothetical protein. conserved | | 1.00 | 1.12 | 1.31 | **1.40** | **1.52** |  |
| Tc00.1047053508741.390 | hypothetical protein. conserved | | 1.09 | 1.19 | 1.32 | 1.25 | **1.38** | Conserved domain: Phospholipase C-like phosphodiesterase |
| Tc00.1047053508879.10 | hypothetical protein. conserved | | -1.31 | -1.16 | **1.80** | 1.02 | 1.06 |  |
| Tc00.1047053508909.160 | hypothetical protein. conserved | | -1.08 | -1.02 | **2.37** | 1.38 | 1.18 |  |
| Tc00.1047053509001.30 | hypothetical protein. conserved | | -1.01 | 1.25 | **1.62** | **1.37** | 1.25 |  |
| Tc00.1047053509029.20 | | hypothetical protein. conserved | 1.32 | **1.98** | **2.13** | **1.79** | **2.67** |  |
| Tc00.1047053509073.60 | | hypothetical protein. conserved | 1.14 | 1.37 | 1.63 | **3.03** | **3.37** |  |
| Tc00.1047053509245.29 | hypothetical protein. conserved | | -1.21 | -1.24 | 1.33 | **1.66** | 1.26 |  |
| Tc00.1047053509393.10 | hypothetical protein. conserved | | -1.02 | 1.26 | **1.58** | 1.41 | 1.22 |  |
| Tc00.1047053509571.30 | hypothetical protein. conserved | | 1.18 | 1.25 | 1.36 | **1.76** | **1.96** |  |
| Tc00.1047053509601.140 | | hypothetical protein | -1.03 | 1.35 | **1.61** | **1.42** | **1.68** |  |
| Tc00.1047053509627.10 | | hypothetical protein. conserved | 1.52 | 1.58 | 1.58 | 1.63 | **1.90** |  |
| Tc00.1047053509755.89 | | hypothetical protein | 1.02 | -1.13 | -1.33 | 1.05 | **1.39** |  |
| Tc00.1047053510173.120 | | hypothetical protein. conserved | 1.66 | 1.74 | **2.16** | **2.24** | **2.19** |  |
| Tc00.1047053510381.40 | hypothetical protein. conserved | | -1.00 | 1.24 | **1.61** | 1.35 | 1.23 |  |
| Tc00.1047053510479.30 | | hypothetical protein | 1.15 | 1.02 | 1.05 | 1.30 | **2.31** |  |
| Tc00.1047053510535.10 | | hypothetical protein. conserved | 1.44 | **2.31** | **2.31** | **2.11** | **2.56** |  |
| Tc00.1047053510599.40 | hypothetical protein. conserved | | 1.19 | 1.22 | 1.26 | **1.43** | **1.48** |  |
| Tc00.1047053510835.20 | hypothetical protein. conserved | | 1.47 | 1.32 | 1.22 | **1.95** | **2.81** |  |
| Tc00.1047053511121.30 | | hypothetical protein | 1.25 | 1.43 | 1.36 | **1.84** | **2.10** |  |
| Tc00.1047053511201.40 | | hypothetical protein. conserved | 1.36 | 1.47 | **1.74** | **2.10** | **2.44** |  |
| Tc00.1047053511239.60 | hypothetical protein. conserved | | 1.06 | 1.21 | 1.14 | 1.48 | **1.81** |  |
| Tc00.1047053511391.110 | hypothetical protein. conserved | | 1.12 | 1.27 | **1.57** | 1.34 | 1.24 | Conserved domain: histone-lysine N-methyltransferase. putative |
| Tc00.1047053511421.90 | hypothetical protein. conserved | | 1.10 | 1.23 | **1.80** | 1.43 | 1.18 |  |
| Tc00.1047053511467.60 | hypothetical protein. conserved | | 1.00 | 1.11 | **1.91** | 1.17 | 1.32 |  |
| Tc00.1047053511517.130 | hypothetical protein. conserved | | -1.05 | 1.02 | **1.43** | 1.25 | **1.53** |  |
| Tc00.1047053511523.50 | hypothetical protein. conserved | | 1.02 | 1.05 | 1.01 | 1.32 | **1.89** |  |
| Tc00.1047053511611.30 | | hypothetical protein | 1.06 | 1.43 | 1.29 | **1.91** | 1.52 |  |
| Tc00.1047053511643.80 | hypothetical protein. conserved (pseudo.) | | 1.17 | 1.08 | 1.03 | 1.21 | **1.38** |  |
| Tc00.1047053511671.140 | | hypothetical protein | 1.93 | 1.42 | 1.49 | **2.85** | 2.23 |  |
| Tc00.1047053511727.220 | hypothetical protein. conserved | | 1.03 | 1.14 | 1.07 | **1.46** | 1.16 |  |
| Tc00.1047053511755.19 | hypothetical protein. conserved | | -1.05 | 1.05 | **1.96** | 1.52 | 1.65 |  |
| Tc00.1047053445777.10 | RHS protein | | -1.03 | 1.20 | 1.20 | 1.29 | **1.33** | On the edge of chromosome TcChr40-P |
| Tc00.1047053508071.90 | RHS protein | | 1.18 | 1.17 | 1.31 | 1.21 | **1.56** | Contig not mapped (Tcruzi_7589) |
| Tc00.1047053509259.180 | RHS protein | | 1.06 | 1.09 | 1.26 | 1.29 | **1.39** | On the edge of chromosome TcChr25-P |
| Tc00.1047053506537.10 | RHS (pseudog.) | | 1.06 | 1.31 | 1.38 | 1.35 | **1.45** | On the edge of chromosome TcChr23-P |
| Tc00.1047053507975.10 | RHS (pseudog.) | | 1.21 | 1.25 | 1.23 | 1.26 | **1.50** | On the edge of chromosome TcChr17-P |
| Tc00.1047053506951.10 | RHS (pseudog.) | | 1.07 | 1.21 | 1.18 | **1.34** | **1.44** | Repetitive region of TcChr24-S |
| Tc00.1047053504099.70 | RHS (pseudog.) | | 1.14 | 1.19 | 1.29 | 1.31 | **1.33** | On the edge of chromosome TcChr26-S |
| Tc00.1047053508483.40 | RHS (pseudog.) | | 1.08 | 1.18 | 1.19 | 1.45 | **1.57** | Contig not mapped (Tcruzi_7746) |
| Tc00.1047053509765.60 | RHS (pseudog.) | | 1.11 | 1.17 | 1.23 | **1.47** | 1.30 | On the edge of chromosome TcChr17-S |
| Tc00.1047053508877.30 | RHS (pseudog.) | | 1.07 | 1.15 | 1.07 | **1.39** | **1.42** | Repetitive region of TcChr41-P |
| Tc00.1047053505915.10 | RHS (pseudog.) | | 1.02 | 1.14 | **1.47** | **1.48** | 1.46 | Contig not mapped (Tcruzi_6735) |
| Tc00.1047053506683.210 | RHS (pseudog.) | | 1.10 | 1.13 | 1.15 | 1.22 | **1.43** | On the edge of chromosome TcChr12-S |
| Tc00.1047053506751.70 | RHS (pseudog.) | | 1.08 | 1.04 | 1.18 | **1.53** | **1.69** | Repetitive region of TcChr41-P |
| Tc00.1047053504285.10 | RHS (pseudog.) | | 1.02 | -1.16 | **1.54** | 1.13 | 1.03 | Contig not mapped (Tcruzi_6244) |
| Tc00.1047053507427.30 | RHS (pseudog.) | | -1.00 | 1.20 | 1.38 | **1.44** | **1.43** | Contig not mapped (Tcruzi_7332) |
| Tc00.1047053507777.30 | RHS (pseudog.) | | -1.06 | -1.12 | **1.61** | 1.02 | 1.05 | On the edge of chromosome TcChr21-P |
| Tc00.1047053506349.83 | RHS (pseudog.) | | 1.12 | 1.20 | 1.17 | **1.49** | **1.54** | Contig not mapped (Tcruzi_6908) |
| Tc00.1047053506129.80 | RHS (pseudog.) | | 1.14 | 1.15 | 1.24 | **1.52** | **1.50** | On the edge of chromosome TcChr6-S |
| Tc00.1047053506561.20 | RHS (pseudog.) | | -1.05 | -1.13 | 1.16 | 1.23 | **1.33** | On the edge of chromosome TcChr5-P |
| Tc00.1047053509559.20 | RHS (pseudog.) | | 1.07 | 1.02 | 1.15 | **1.34** | **1.40** | Repetitive region of TcChr12-P |
| Tc00.1047053413293.30 | RHS (pseudog.) | | 1.27 | 1.05 | 1.22 | 1.16 | **1.32** | Contig not mapped (Tcruzi_12248) |
| 01404 (Slide ID) | Obsolete | | -1.16 | 1.11 | 1.05 | **1.45** | **1.76** | Repetitive |
| 01497 (Slide ID) | Obsolete | | -1.05 | -1.06 | 1.04 | **1.58** | 1.22 | Repetitive |
| 02117 (Slide ID) | Obsolete | | 1.12 | 1.31 | 1.23 | 1.32 | **1.71** | Inter-CDS |
| 05737 (Slide ID) | Obsolete | | 1.17 | 1.14 | **1.61** | **4.04** | **7.53** | kDNA |
| 05884 (Slide ID) | Obsolete | | -1.10 | 1.37 | 1.56 | **2.30** | **3.15** | Inter-CDS |
| 06361 (Slide ID) | Obsolete | | 1.36 | **1.43** | 1.02 | 1.07 | 1.05 | Not localized |
| 06364 (Slide ID) | Obsolete | | 1.01 | **1.71** | **2.48** | **1.93** | **1.71** | Not localized |
| 07785 (Slide ID) | Obsolete | | 1.06 | 1.34 | 1.47 | **1.88** | **3.20** | Inter-CDS |
| 08041 (Slide ID) | Obsolete | | 1.49 | 1.16 | 1.51 | **3.83** | **4.84** | kDNA |
| 09204 (Slide ID) | Obsolete | | 1.27 | 1.11 | 1.35 | 1.66 | **2.97** | Inter-CDS |
| 10153 (Slide ID) | Obsolete | | 1.27 | 1.28 | 1.46 | **2.64** | **3.63** | kDNA |
| 11281 (Slide ID) | Obsolete | | -1.01 | 1.20 | 1.07 | **1.41** | **2.27** | Not localized |
| 11747 (Slide ID) | Obsolete | | 1.10 | **1.81** | **2.20** | **1.86** | **2.28** | Inter-CDS |
| 13664 (Slide ID) | Obsolete | | 1.09 | 1.10 | **2.18** | 1.10 | -1.04 | Inter-CDS |
| 13828 (Slide ID) | Obsolete | | 1.36 | **1.70** | 1.34 | 1.27 | 1.52 | Inter-CDS |
| 13999 (Slide ID) | Obsolete | | 1.16 | 1.38 | 1.46 | **1.58** | 1.44 | Inside a not mapped contig |
| 14569 (Slide ID) | Obsolete | | 1.74 | 1.30 | 1.65 | **3.71** | 1.53 | kDNA |
| 14862 (Slide ID) | Obsolete | | 1.66 | **1.97** | 1.50 | **1.97** | **2.24** | Inter-CDS |
| 16329 (Slide ID) | Obsolete | | 1.28 | **1.41** | **1.45** | **1.39** | 1.03 | Inter-CDS |
| 18649 (Slide ID) | Obsolete | | 1.17 | 1.14 | 1.23 | 1.42 | **1.82** | Repetitive |
| 18850 (Slide ID) | Obsolete | | -1.03 | 1.34 | **1.80** | 1.08 | 1.08 | Inter-CDS |
| 19423 (Slide ID) | Obsolete | | 1.07 | 1.28 | 1.57 | 1.52 | **1.70** | Inter-CDS |
| 21055 (Slide ID) | Obsolete | | 1.20 | 1.33 | 1.29 | 1.22 | **1.40** | Inter-CDS |
| 21056 (Slide ID) | Obsolete | | 1.18 | 1.18 | 1.28 | 1.25 | **1.70** | Inter-CDS |
| 21269 (Slide ID) | Obsolete | | 1.14 | 1.09 | **1.70** | -1.13 | 1.06 | Inside a not mapped contig |
| 21387 (Slide ID) | Obsolete | | 1.66 | **1.97** | 1.50 | **1.97** | **2.24** | Inter-CDS |
| 23196 (Slide ID) | Obsolete | | 1.15 | **1.59** | **1.63** | 1.43 | **1.90** | Inter-CDS |
| 24439 (Slide ID) | Obsolete | | 1.10 | **1.81** | **2.20** | **1.86** | **2.28** | Inter-CDS |
| 26458 (Slide ID) | Obsolete | | **1.44** | 1.23 | 1.10 | -1.15 | **1.44** | Inter-CDS |

Bolded fold-changes: significant values. i.a.i.: immediately after irradiation. Additional information is available when appropriate.
