# Supplementary material for: Trypanosoma cruzi Gene Expression in Response to Gamma Radiation
Source: PLoS One. 2012 Jan 11;7(1):e29596. doi: 10.1371/journal.pone.0029596 (PMC3256153; doi:10.1371/journal.pone.0029596)
Supplement: Table S3 — GO-Slim terms for Biological Processes (A and B) and Molecular Function (C and D) categories and their respective genes. (DOC) [file pone.0029596.s006.doc]

| ID | GO-Slim Name | Cluster | Sequence ID | Product |
| --- | --- | --- | --- | --- |
| 1. **Biological Process – Down-regulated genes** | | | | |
| GO:0005975 | Carbohydrate metabolic process | 12 | Tc00.1047053510091.80 | pyruvate dehydrogenase E1 beta subunit. putative |
|  |  | 12 | Tc00.1047053511041.40 | hexose transporter. putative |
|  |  | 12 | Tc00.1047053507547.90 | glycosomal phosphoenolpyruvate carboxykinase. putative |
| GO:0006091 | Generation of precursor metabolites and energy | 12 | Tc00.1047053503929.10 | V-type ATPase. A subunit. putative |
|  |  | 11 | Tc00.1047053508479.340 | succinyl-CoA synthetase alpha subunit. putative |
|  |  | 10 | Tc00.1047053509769.60 | hypothetical protein. conserved (2-iron 2-sulfer protein. component of succinate dehydrogenase complex) |
| GO:0006139 | Nucleobase. nucleoside. nucleotide and nucleic acid metabolic process | 12 | Tc00.1047053506773.50 | nucleoside transporter-like. putative |
|  |  | 11 | Tc00.1047053508707.200 | nucleoside diphosphate kinase. putative |
|  |  | 12 | Tc00.1047053506855.260 | thymidine kinase. putative |
| GO:0006350 | Transcription | 10 | Tc00.1047053510645.20 | nuclear transcription factor. putative |
| GO:0006412 | Translation | 12 | Tc00.1047053507671.30 | 25 kDa translation elongation factor 1-beta |
|  |  | 11 | Tc00.1047053510101.430 | 40S ribosomal protein S21. putative |
|  |  | 11 | Tc00.1047053509353.30 | 40S ribosomal protein S3. putative |
|  |  | 11 | Tc00.1047053506297.150 | 40S ribosomal protein S5. putative |
|  |  | 11 | Tc00.1047053510425.19 | 40S ribosomal protein SA. putative |
|  |  | 11 | Tc00.1047053503719.20 | 40S ribosomal protein SA. putative |
|  |  | 11 | Tc00.1047053505977.26 | 60S acidic ribosomal protein P2. putative |
|  |  | 11 | Tc00.1047053508461.490 | 60S ribosomal protein L23. putative |
|  |  | 11 | Tc00.1047053506297.270 | 60S ribosomal protein L28. putative |
|  |  | 12 | Tc00.1047053510119.9 | elongation factor 1-alpha (ef-1-alpha). putative |
|  |  | 10 | Tc00.1047053508153.730 | elongation initiation factor 2 alpha subunit. putative |
|  |  | 10 | Tc00.1047053463269.10 | eukaryotic translation initiation factor 1A. putative |
|  |  | 9 | Tc00.1047053506943.160 | eukaryotic translation initiation factor 3 subunit 7-like protein. putative |
|  |  | 12 | Tc00.1047053506679.70 | eukaryotic translation initiation factor 6 (eIF-6). putative |
|  |  | 11 | Tc00.1047053507251.20 | ribosomal protein L21E (60S). putative |
|  |  | 12 | Tc00.1047053508421.30 | tryptophanyl-tRNA synthetase (pseudogene). putative |
| GO:0006464 | Protein modification process | 9 | Tc00.1047053503487.50 | UDP-Gal or UDP-GlcNAc-dependent glycosyltransferase. putative |
|  |  | 12 | Tc00.1047053507509.40 | hypothetical protein. conserved (kinase protein) |
| GO:0006629 | Lipid metabolic process | 9 | Tc00.1047053504427.70 | 3-oxo-5-alpha-steroid 4-dehydrogenase. putative |
|  |  | 12 | Tc00.1047053511823.70 | Farnesyl diphosphate synthase |
|  |  | 12 | Tc00.1047053511075.9 | fatty acid desaturase. putative |
|  |  | 10 | Tc00.1047053436521.9 | mevalonate kinase. putative |
|  |  | 12 | Tc00.1047053507617.9 | prostaglandin F2alpha synthase |
|  |  | 12 | Tc00.1047053508461.80 | prostaglandin F2alpha synthase |
|  |  | 10 | Tc00.1047053511071.50 | hypothetical protein. conserved (pseudogene) (HMG-CoA synthase) |
| GO:0006810 | Transport | 10 | Tc00.1047053510187.270 | COP-coated vesicle membrane protein erv25 precursor. putative.ER--golgi transport protein erv25 precursor. putative |
|  |  | 12 | Tc00.1047053503539.30 | GTP-binding nuclear protein rtb2. putative |
|  |  | 12 | Tc00.1047053511041.40 | hexose transporter. putative |
|  |  | 9 | Tc00.1047053510667.14 | membrane transporter protein. putative |
|  |  | 10 | Tc00.1047053508173.180 | nuclear transport factor 2 protein(NFT2). putative |
|  |  | 12 | Tc00.1047053506773.50 | nucleoside transporter-like. putative |
|  |  | 12 | Tc00.1047053503929.10 | V-type ATPase. A subunit. putative |
|  |  | 11 | Tc00.1047053507993.380 | hypothetical protein. conserved (signal recognition particle component) |
| GO:0006950 | Response to stress | 12 | Tc00.1047053507641.280 | chaperonin Hsp60. mitochondrial precursor. groELprotein. heat shock protein 60 |
|  |  | 11 | Tc00.1047053510187.420 | chaperonin HSP60. mitochondrial precursor (pseudogene). groELprotein |
|  |  | 11 | Tc00.1047053508173.100 | Monooxygenase. putative |
|  |  | 10 | Tc00.1047053503555.30 | trypanothione reductase. putative |
|  |  | 11 | Tc00.1047053511745.10 | heat shock 70 kDa protein. mitochondrial precursor. putative |
| GO:0007165 | Signal transduction | 12 | Tc00.1047053503539.30 | GTP-binding nuclear protein rtb2. putative |
| GO:0016043 | Cellular component organization | 12 | Tc00.1047053411235.9 | alpha tubulin. putative |
|  |  | 12 | Tc00.1047053506563.40 | beta tubulin. putative |
|  |  | 12 | Tc00.1047053506679.70 | eukaryotic translation initiation factor 6 (eIF-6). putative |
|  |  | 10 | Tc00.1047053507943.40 | histone H4. putative |
| GO:0019538 | Protein metabolic process | 11 | Tc00.1047053504221.20 | 26S proteasome regulatory non-ATPase subunit. putative |
|  |  | 12 | Tc00.1047053510655.120 | aminopeptidase P. putative. metallo-peptidase. Clan MG. Family M24. putative |
|  |  | 12 | Tc00.1047053504153.160 | carboxypeptidase. putative. metallo-peptidase. clan MA(E). family M32. putative |
|  |  | 12 | Tc00.1047053507641.280 | chaperonin Hsp60. mitochondrial precursor. groELprotein. heat shock protein 60 |
|  |  | 11 | Tc00.1047053510187.420 | chaperonin HSP60. mitochondrial precursor (pseudogene). groELprotein. degenerate.heat shock protein 60. degenerate. chaperonin HSP60. mitochondrial precursor. degenerate |
|  |  | 12 | Tc00.1047053506247.50 | chaperonin. putative. T-complex protein 1. theta subunit. putative |
|  |  | 12 | Tc00.1047053506529.550 | cysteine peptidase. putative. cysteine peptidase. clan CA. family C1. cathepsin L-like. putative |
|  |  | 12 | Tc00.1047053508317.10 | cysteine proteinase. putative |
|  |  | 11 | Tc00.1047053511745.10 | heat shock 70 kDa protein. mitochondrial precursor. putative |
|  |  | 10 | Tc00.1047053509267.40 | hypothetical protein. conserved (ARM motif) |
| **B) Biological Process – Up-regulated genes** | | | | |
| GO:0005975 | Carbohydrate metabolic process | 4 | Tc00.1047053506529.508 | glucose-6-phosphate isomerase. glycosomal. putative |
| GO:0006139 | Nucleobase. nucleoside. nucleotide and nucleic acid metabolic process | 3 | Tc00.1047053508375.30 | aspartate carbamoyltransferase. putative |
|  |  | 1 | Tc00.1047053508461.400 | nucleoside diphosphate kinase. putative |
|  |  | 1 | Tc00.1047053507611.290 | acetyltransferase. putative |
|  |  | 4 | Tc00.1047053507083.10 | hypothetical protein. conserved |
| GO:0006281 | DNA repair | 3 | Tc00.1047053506619.40 | tyrosyl-DNA Phosphodiesterase (Tdp1). putative |
| GO:0006464 | Protein modification process | 1 | Tc00.1047053510121.130 | serine/threonine protein kinase. putative |
|  |  | 1 | Tc00.1047053507611.290 | acetyltransferase. putative |
|  |  | 1 | Tc00.1047053511269.50 | protein kinase A catalytic subunit. putative |
|  |  | 1 | Tc00.1047053504113.10 | protein kinase. putative |
|  |  | 1 | Tc00.1047053511671.80 | protein kinase. putative |
| GO:0006519 | cellular amino acid and derivative metabolic process | 3 | Tc00.1047053508375.30 | aspartate carbamoyltransferase. putative |
|  |  | 1 | Tc00.1047053508457.30 | glycine cleavage system H protein. putative |
| GO:0006629 | lipid metabolic process | 4 | Tc00.1047053473111.10 | lathosterol oxidase. putative |
|  |  | 1 | Tc00.1047053508741.390 | hypothetical protein. conserved (Phospholipase C-like phosphodiesterase) |
| GO:0006810 | transport | 1 | Tc00.1047053508357.80 | cation transporter protein. putative |
|  |  | 1 | Tc00.1047053506947.50 | hypothetical protein. conserved (major facilitator superfamily conserved domain) |
| GO:0006950 | response to stress | 3 | Tc00.1047053506619.40 | tyrosyl-DNA Phosphodiesterase (Tdp1). putative |
|  |  | 2 | Tc00.1047053508649.5 | tryparedoxin peroxidase. putative |
|  |  | 3 | Tc00.1047053507929.20 | co-chaperone GrpE. putative |
|  |  | 7 | Tc00.1047053503899.119 | trypanothione/tryparedoxin dependent peroxidase 2. putative |
| GO:0007049 | cell cycle | 1 | Tc00.1047053509455.140 | cyclin. putative |
| GO:0009405 | pathogenesis | 1 | Tc00.1047053510199.10 | GP85-like protein. putative |
|  |  | 1 | Tc00.1047053509907.60 | trans-sialidase (pseudogene). putative |
| GO:0009058 | biosynthetic process | 1 | Tc00.1047053507083.70 | hypothetical protein. conserved |
| GO:0016043 | cellular component organization | 2 | Tc00.1047053508737.194 | ARP2/3 complex subunit. putative |
|  |  | 4 | Tc00.1047053509069.30 | tubulin binding cofactor A-like protein. putative |
| GO:0019538 | protein metabolic process | 3 | Tc00.1047053508183.4 | aspartyl aminopeptidase. putative |
|  |  | 2 | Tc00.1047053506563.210 | calpain-like cysteine peptidase. putative. cysteine peptidase. Clan CA. family C2. putative |
|  |  | 3 | Tc00.1047053507929.20 | co-chaperone GrpE. putative |
| GO:0016193 | vesicle-mediated transport | 3 | Tc00.1047053508479.290 | vacuolar sorting protein. putative |
| **C) Molecular Function – Down-regulated genes** | | | | |
| GO:0005524 | ATP binding | 12 | Tc00.1047053507641.280 | chaperonin Hsp60. mitochondrial precursor. groELprotein. heat shock protein 60 |
|  |  | 11 | Tc00.1047053510187.420 | chaperonin HSP60. mitochondrial precursor (pseudogene).groELprotein. degenerate.heat shock protein 60. degenerate.chaperonin HSP60. mitochondrial precursor. degenerate |
|  |  | 12 | Tc00.1047053506247.50 | chaperonin. putative. T-complex protein 1. theta subunit. putative |
|  |  | 12 | Tc00.1047053507547.90 | glycosomal phosphoenolpyruvate carboxykinase. putative |
|  |  | 10 | Tc00.1047053436521.9 | mevalonate kinase. putative |
|  |  | 10 | Tc00.1047053510155.20 | mitochondrial RNA editing ligase 1. putative |
|  |  | 11 | Tc00.1047053508707.200 | nucleoside diphosphate kinase. putative |
|  |  | 12 | Tc00.1047053506855.260 | thymidine kinase. putative |
|  |  | 12 | Tc00.1047053508421.30 | tryptophanyl-tRNA synthetase (pseudogene). putative |
|  |  | 12 | Tc00.1047053503929.10 | V-type ATPase. A subunit. putative |
|  |  | 11 | Tc00.1047053511745.10 | heat shock 70 kDa protein. mitochondrial precursor. putative |
|  |  | 12 | Tc00.1047053507509.40 | hypothetical protein. conserved (kinase protein) |
| GO:0005526 | GTP binding | 12 | Tc00.1047053411235.9 | alpha tubulin. putative |
|  |  | 12 | Tc00.1047053510119.9 | elongation factor 1-alpha (ef-1-alpha). putative |
|  |  | 12 | Tc00.1047053503539.30 | GTP-binding nuclear protein rtb2. putative |
|  |  | 12 | Tc00.1047053506563.40 | beta tubulin. putative |
| GO:0010181 | FMN/FAD binding | 12 | Tc00.1047053507617.9 | prostaglandin F2alpha synthase |
|  |  | 10 | Tc00.1047053503555.30 | trypanothione reductase. putative |
|  |  | 12 | Tc00.1047053508461.80 | prostaglandin F2alpha synthase |
| GO:0003677 | DNA binding | 10 | Tc00.1047053507943.40 | histone H4. putative |
|  |  | 9 | Tc00.1047053509793.10 | kinetoplast DNA-associated protein. putative |
| GO:0008270 | Zinc ion binding | 10 | Tc00.1047053510645.20 | nuclear transcription factor. putative |
|  |  | 12 | Tc00.1047053509669.40 | Zn-finger protein. putative |
| GO:0003723 | RNA binding | 12 | Tc00.1047053510859.17 | nucleolar RNA-binding protein. putative |
|  |  | 11 | Tc00.1047053509353.30 | 40S ribosomal protein S3. putative |
|  |  | 11 | Tc00.1047053506297.150 | 40S ribosomal protein S5. putative |
|  |  | 10 | Tc00.1047053508153.730 | elongation initiation factor 2 alpha subunit. putative |
|  |  | 10 | Tc00.1047053463269.10 | eukaryotic translation initiation factor 1A. putative |
|  |  | 9 | Tc00.1047053511715.100 | pumilio/PUF RNA binding protein 7. putative |
|  |  | 11 | Tc00.1047053507993.380 | hypothetical protein. conserved (signal recognition particle component) |
| GO:0003824 | Catalytic activity | 11 | Tc00.1047053510099.120 | D-isomer specific 2-hydroxyacid dehydrogenase-protein. putative |
|  |  | 11 | Tc00.1047053510131.40 | haloacid dehalogenase-like hydrolase. putative |
|  |  | 9 | Tc00.1047053511355.30 | phosphatidic acid phosphatase protein. putative |
|  |  | 11 | Tc00.1047053508479.340 | succinyl-CoA synthetase alpha subunit. putative |
|  |  | 10 | Tc00.1047053511071.50 | hypothetical protein. conserved (pseudogene) (HMG-CoA synthase) |
| GO:0016491 | Oxidoreductase activity | 9 | Tc00.1047053504427.70 | 3-oxo-5-alpha-steroid 4-dehydrogenase. putative |
|  |  | 12 | Tc00.1047053511391.160 | cytochrome c1. heme protein. mitochondrial precursor. putative |
|  |  | 11 | Tc00.1047053510099.120 | D-isomer specific 2-hydroxyacid dehydrogenase-protein. putative |
|  |  | 12 | Tc00.1047053511075.9 | fatty acid desaturase. putative |
|  |  | 11 | Tc00.1047053508173.100 | Monooxygenase. putative |
|  |  | 12 | Tc00.1047053511817.40 | NADH-cytochrome B5 reductase. putative |
|  |  | 12 | Tc00.1047053507617.9 | prostaglandin F2alpha synthase |
|  |  | 12 | Tc00.1047053508461.80 | prostaglandin F2alpha synthase |
|  |  | 12 | Tc00.1047053510091.80 | pyruvate dehydrogenase E1 beta subunit. putative |
|  |  | 10 | Tc00.1047053503555.30 | trypanothione reductase. putative |
|  |  | 10 | Tc00.1047053509769.60 | hypothetical protein. conserved (2-iron 2-sulfer protein. component of succinate dehydrogenase complex) |
|  |  | 12 | Tc00.1047053506219.40 | hypothetical protein. conserved (short chain dehydrogenase) |
| GO:0016874 | ligase activity | 10 | Tc00.1047053510155.20 | mitochondrial RNA editing ligase 1. putative |
|  |  | 12 | Tc00.1047053508421.30 | tryptophanyl-tRNA synthetase (pseudogene). putative |
| GO:0004672 | protein kinase activity | 12 | Tc00.1047053507509.40 | hypothetical protein. conserved (kinase protein) |
|  |  | 11 | Tc00.1047053511211.120 | activated protein kinase C receptor. putative. guanine nucleotide-binding protein beta subunit-like protein. putative |
| GO:0003735 | structural constituent of ribosome | 11 | Tc00.1047053510101.430 | 40S ribosomal protein S21. putative |
|  |  | 11 | Tc00.1047053509353.30 | 40S ribosomal protein S3. putative |
|  |  | 11 | Tc00.1047053506297.150 | 40S ribosomal protein S5. putative |
|  |  | 11 | Tc00.1047053510425.19 | 40S ribosomal protein SA. putative |
|  |  | 11 | Tc00.1047053503719.20 | 40S ribosomal protein SA. putative |
|  |  | 11 | Tc00.1047053505977.26 | 60S acidic ribosomal protein P2. putative |
|  |  | 11 | Tc00.1047053507251.20 | 60S ribosomal protein L21E. putative |
|  |  | 11 | Tc00.1047053508461.490 | 60S ribosomal protein L23. putative |
|  |  | 11 | Tc00.1047053506297.270 | 60S ribosomal protein L28. putative |
| GO:0005198 | Structural molecule activity | 12 | Tc00.1047053411235.9 | alpha tubulin. putative |
|  |  | 12 | Tc00.1047053506563.40 | beta tubulin. putative |
| GO:0005215 | transporter activity | 12 | Tc00.1047053511391.160 | cytochrome c1. heme protein. mitochondrial precursor. putative |
|  |  | 12 | Tc00.1047053511041.40 | hexose transporter. putative |
|  |  | 9 | Tc00.1047053510667.14 | membrane transporter protein. putative |
|  |  | 12 | Tc00.1047053506773.50 | nucleoside transporter-like. putative |
|  |  | 12 | Tc00.1047053503929.10 | V-type ATPase. A subunit. putative |
| GO:0005509 | calcium ion binding | 8 | Tc00.1047053507891.47 | flagellar calcium-binding protein. putative |
| GO:0005515 | protein binding | 12 | Tc00.1047053507641.280 | chaperonin Hsp60. mitochondrial precursor. groELprotein. heat shock protein 60 |
|  |  | 11 | Tc00.1047053507873.20 | cell differentiation protein. putative |
|  |  | 11 | Tc00.1047053510187.420 | chaperonin HSP60. mitochondrial precursor (pseudogene).groELprotein. degenerate.heat shock protein 60. degenerate.chaperonin HSP60. mitochondrial precursor. degenerate |
|  |  | 12 | Tc00.1047053506247.50 | chaperonin. putative. T-complex protein 1. theta subunit. putative |
|  |  | 12 | Tc00.1047053503539.30 | GTP-binding nuclear protein rtb2. putative |
|  |  | 11 | Tc00.1047053511745.10 | heat shock 70 kDa protein. mitochondrial precursor. putative |
|  |  | 10 | Tc00.1047053509267.40 | hypothetical protein. conserved (ARM motif) |
| GO:0008135 | translation factor activity. nucleic acid binding | 12 | Tc00.1047053507671.30 | 25 kDa translation elongation factor 1-beta |
|  |  | 12 | Tc00.1047053510119.9 | elongation factor 1-alpha (ef-1-alpha). putative |
|  |  | 10 | Tc00.1047053508153.730 | elongation initiation factor 2 alpha subunit. putative |
|  |  | 10 | Tc00.1047053463269.10 | eukaryotic translation initiation factor 1A. putative |
|  |  | 9 | Tc00.1047053506943.160 | eukaryotic translation initiation factor 3 subunit 7-like protein. putative |
|  |  | 12 | Tc00.1047053506679.70 | eukaryotic translation initiation factor 6 (eIF-6). putative |
| GO:0008233 | peptidase activity | 12 | Tc00.1047053510655.120 | aminopeptidase P. putative.metallo-peptidase. Clan MG. Family M24. putative |
|  |  | 12 | Tc00.1047053504153.160 | carboxypeptidase. putative.metallo-peptidase. clan MA(E). family M32. putative |
|  |  | 12 | Tc00.1047053506529.550 | cysteine peptidase. putative. cysteine peptidase. clan CA. family C1. cathepsin L-like. putative |
|  |  | 12 | Tc00.1047053508317.10 | cysteine proteinase. putative |
| GO:0009055 | electron carrier activity | 12 | Tc00.1047053511391.160 | cytochrome c1. heme protein. mitochondrial precursor. putative |
|  |  | 10 | Tc00.1047053509769.60 | hypothetical protein. conserved (2-iron 2-sulfer protein. component of succinate dehydrogenase complex) |
| GO:0016301 | kinase activity | 12 | Tc00.1047053507547.90 | glycosomal phosphoenolpyruvate carboxykinase. putative |
|  |  | 10 | Tc00.1047053436521.9 | mevalonate kinase. putative |
|  |  | 11 | Tc00.1047053508707.200 | nucleoside diphosphate kinase. putative |
|  |  | 12 | Tc00.1047053506855.260 | thymidine kinase. putative |
| GO:0016740 | transferase activity | 12 | Tc00.1047053509693.100 | 2-aminoethylphosphonate:pyruvateaminotransferase- likeprotein.putative |
|  |  | 12 | Tc00.1047053511823.70 | Farnesyl diphosphate synthase |
|  |  | 9 | Tc00.1047053503487.50 | UDP-Gal or UDP-GlcNAc-dependent glycosyltransferase. putative |
|  |  | 12 | Tc00.1047053507509.40 | hypothetical protein. conserved (kinase protein) |
|  |  | 10 | Tc00.1047053511071.50 | hypothetical protein. conserved (pseudogene) (HMG-CoA synthase) |
| GO:0016787 | hydrolase activity | 12 | Tc00.1047053411235.9 | alpha tubulin. putative |
|  |  | 12 | Tc00.1047053510655.120 | aminopeptidase P. putative.metallo-peptidase. Clan MG. Family M24. putative |
|  |  | 12 | Tc00.1047053506563.40 | beta tubulin. putative |
|  |  | 12 | Tc00.1047053504153.160 | carboxypeptidase. putative.metallo-peptidase. clan MA(E). family M32. putative |
|  |  | 12 | Tc00.1047053506529.550 | cysteine peptidase. putative. cysteine peptidase. clan CA. family C1. cathepsin L-like. putative |
|  |  | 12 | Tc00.1047053508317.10 | cysteine proteinase. putative |
|  |  | 12 | Tc00.1047053510119.9 | elongation factor 1-alpha (ef-1-alpha). putative |
|  |  | 12 | Tc00.1047053503539.30 | GTP-binding nuclear protein rtb2. putative |
|  |  | 11 | Tc00.1047053510131.40 | haloacid dehalogenase-like hydrolase. putative |
|  |  | 12 | Tc00.1047053503929.10 | V-type ATPase. A subunit. putative |
| GO:0030234 | enzyme regulator activity | 11 | Tc00.1047053504221.20 | 26S proteasome regulatory non-ATPase subunit. putative |
| **D) Molecular Function - Up-regulated genes** | | | | |
| GO:0010181/GO:0050660 | FMN/FAD binding | 2 | Tc00.1047053509941.100 | 2.4-dienoyl-coa reductase fadh1. putative |
| GO:0005524 | ATP binding | 7 | Tc00.1047053504149.20 | ATP-binding cassette transporter ABCA1. putative |
|  |  | 2 | Tc00.1047053511421.110 | developmentally regulated phosphoprotein. putative |
|  |  | 1 | Tc00.1047053510687.10 | dynein heavy chain (pseudogene). putative |
|  |  | 1 | Tc00.1047053509585.10 | dynein heavy chain. putative |
|  |  | 1 | Tc00.1047053508461.400 | nucleoside diphosphate kinase. putative |
|  |  | 1 | Tc00.1047053510121.130 | serine/threonine protein kinase. putative |
|  |  | 1 | Tc00.1047053511269.50 | protein kinase A catalytic subunit. putative |
|  |  | 1 | Tc00.1047053504113.10 | protein kinase. putative |
|  |  | 1 | Tc00.1047053511671.80 | protein kinase. putative |
| GO:0000166 | Nucleotide binding | 1 | Tc00.1047053510105.100 | UDP-glucose dehydrogenase. putative |
|  |  | 3 | Tc00.1047053507929.20 | co-chaperone GrpE. putative |
| GO:0003774 | Motor activity | 1 | Tc00.1047053510687.10 | dynein heavy chain (pseudogene). putative |
|  |  | 1 | Tc00.1047053509585.10 | dynein heavy chain. putative |
|  |  | 1 | Tc00.1047053510897.6 | Dynein light chain LC6. flagellar outer arm. putative |
| GO:0016491 | Oxidoreductase activity | 2 | Tc00.1047053509941.100 | 2.4-dienoyl-coa reductase fadh1. putative |
|  |  | 4 | Tc00.1047053473111.10 | lathosterol oxidase. putative |
|  |  | 1 | Tc00.1047053510105.100 | UDP-glucose dehydrogenase. putative |
|  |  | 7 | Tc00.1047053503899.119 | trypanothione/tryparedoxin dependent peroxidase 2. putative |
|  |  | 2 | Tc00.1047053508649.5 | tryparedoxin peroxidase. putative |
|  |  | 3 | Tc00.1047053506357.50 | alcohol dehydrogenase. putative |
| GO:0016829 | Lyase activity | 2 | Tc00.1047053510659.240 | lactoylglutathione lyase-like protein. putative |
|  |  | 3 | Tc00.1047053510743.70 | lactoylglutathione lyase-like protein. putative |
| GO:0003824 | Catalytic activity | 3 | Tc00.1047053503841.20 | hypothetical protein. conserved (CBS conserved domain) |
| GO:0004672 | Protein kinase activity | 1 | Tc00.1047053510121.130 | serine/threonine protein kinase. putative |
|  |  | 1 | Tc00.1047053511269.50 | protein kinase A catalytic subunit. putative |
|  |  | 1 | Tc00.1047053504113.10 | protein kinase. putative |
|  |  | 1 | Tc00.1047053511671.80 | protein kinase. putative |
| GO:0005215 | Transporter activity | 1 | Tc00.1047053508357.80 | cation transporter protein. putative |
|  |  | 3 | Tc00.1047053508479.290 | vacuolar sorting protein. putative |
|  |  | 1 | Tc00.1047053506947.50 | hypothetical protein. conserved (major facilitator superfamily conserved domain) |
| GO:0051536 | Iron-sulfur cluster binding | 1 | Tc00.1047053507083.70 | hypothetical protein. conserved |
| GO:0008270 | Zinc ion binding | 3 | Tc00.1047053508183.4 | aspartyl aminopeptidase. putative |
|  |  | 2 | Tc00.1047053506739.99 | hypothetical protein. conserved |
|  |  | 3 | Tc00.1047053503839.19 | hypothetical protein. conserved (B-box zinc-finger conserved domain) |
| GO:0005509 | Calcium ion binding | 2 | Tc00.1047053506391.30 | ef-hand protein 5. putative |
|  |  | 2 | Tc00.1047053507483.20 | ef-hand protein 5. putative |
|  |  | 1 | Tc00.1047053507165.30 | hypothetical protein. conserved (EF-hand conserved domain) |
| GO:0005515 | Protein binding | 4 | Tc00.1047053509069.30 | tubulin binding cofactor A-like protein. putative |
|  |  | 2 | Tc00.1047053508215.9 | protein kinase C substrate protein. heavy chain. putative |
|  |  | 3 | Tc00.1047053507929.20 | co-chaperone GrpE. putative |
|  |  | 2 | Tc00.1047053508059.50 | hypothetical protein. conserved |
| GO:0008233 | Peptidase activity | 3 | Tc00.1047053508183.4 | aspartyl aminopeptidase. putative |
|  |  | 2 | Tc00.1047053506563.210 | calpain-like cysteine peptidase. putative. cysteine peptidase. Clan CA. family C2. putative |
| GO:0016301 | Kinase activity | 1 | Tc00.1047053508461.400 | nucleoside diphosphate kinase. putative |
|  |  | 1 | Tc00.1047053510121.130 | serine/threonine protein kinase. putative |
|  |  | 1 | Tc00.1047053511269.50 | protein kinase A catalytic subunit. putative |
|  |  | 2 | Tc00.1047053508215.9 | protein kinase C substrate protein. heavy chain. putative |
|  |  | 1 | Tc00.1047053504113.10 | protein kinase. putative |
|  |  | 1 | Tc00.1047053511671.80 | protein kinase. putative |
| GO:0016740 | Transferase activity | 3 | Tc00.1047053508375.30 | aspartate carbamoyltransferase. putative |
|  |  | 1 | Tc00.1047053508457.30 | glycine cleavage system H protein. putative |
|  |  | 1 | Tc00.1047053508461.400 | nucleoside diphosphate kinase. putative |
|  |  | 1 | Tc00.1047053511269.50 | protein kinase A catalytic subunit. putative |
|  |  | 1 | Tc00.1047053504113.10 | protein kinase. putative |
|  |  | 1 | Tc00.1047053511671.80 | protein kinase. putative |
|  |  | 1 | Tc00.1047053507611.290 | acetyltransferase. putative |
|  |  | 2 | Tc00.1047053511391.110 | hypothetical protein. conserved (histone-lysine N-methyltransferase. putative) |
| GO:0016787 | Hydrolase activity | 3 | Tc00.1047053508183.4 | aspartyl aminopeptidase. putative |
|  |  | 7 | Tc00.1047053504149.20 | ATP-binding cassette transporter ABCA1. putative |
|  |  | 2 | Tc00.1047053506563.210 | calpain-like cysteine peptidase. putative. cysteine peptidase. Clan CA. family C2. putative |
|  |  | 1 | Tc00.1047053510687.10 | dynein heavy chain (pseudogene). putative |
|  |  | 1 | Tc00.1047053509585.10 | dynein heavy chain. putative |
|  |  | 1 | Tc00.1047053510199.10 | GP85-like protein. putative |
|  |  | 3 | Tc00.1047053506619.40 | tyrosyl-DNA Phosphodiesterase (Tdp1). putative |
|  |  | 1 | Tc00.1047053509907.60 | trans-sialidase (pseudogene). putative |
|  |  | 7 | Tc00.1047053506341.10 | N-acetylglucosamine-6-phosphate deacetylase-like protein. putative |
|  |  | 1 | Tc00.1047053508741.390 | hypothetical protein. conserved ( (Phospholipase C-like phosphodiesterase) |
| GO:0030234 | Enzyme regulator activity | 3 | Tc00.1047053507929.20 | co-chaperone GrpE. putative |

A and C: down-regulated genes. B and D: up-regulated genes. Cluster column indicates the cluster number as in Fig. 7B.
