# Supplementary material for: Trypanosoma cruzi Gene Expression in Response to Gamma Radiation
Source: PLoS One. 2012 Jan 11;7(1):e29596. doi: 10.1371/journal.pone.0029596 (PMC3256153; doi:10.1371/journal.pone.0029596)
Supplement: Table S4 — Gene composition of each heatmap cluster. (DOC) [file pone.0029596.s007.doc]

| **#** | **Genes (with ID number)** |
| --- | --- |
| Cluster 1 | **GP85** (Tc00.1047053510199.10). **acetyltransferase** (Tc00.1047053507611.290). **ATPase. putative** (Tc00.1047053505997.70 e Tc00.1047053508903.100). **calpain-like cysteine peptidase (pseudogene)** (Tc00.1047053506563.170). **cation transporter protein. putative** (Tc00.1047053508357.80). **cyclin. putative** (Tc00.1047053509455.140). **dynein heavy chain** (Tc00.1047053509585.10 e Tc00.1047053510687.10). **dynein light chain (lc6)** (Tc00.1047053510897.6). **glycine cleavage system H protein. putative.** (Tc00.1047053508457.30**). nucleoside diphosphate kinase. putative** (Tc00.1047053508461.400). **protein kinase. putative** (Tc00.1047053504113.10 e Tc00.1047053511671.80). **protein kinase A catalytic subunit. putative** (Tc00.1047053511269.50). **serine/threonine protein kinase. putative** (Tc00.1047053510121.130). **trans-sialidase** (Tc00.1047053509907.60). **UDP-glucose dehydrogenase. putative** (Tc00.1047053510105.100). **hypothetical proteins** (Tc00.1047053511121.30. Tc00.1047053508061.10. Tc00.1047053508293.130. Tc00.1047053510479.30. Tc00.1047053511611.30. Tc00.1047053506133.179. Tc00.1047053507521.60. Tc00.1047053509571.30. Tc00.1047053510599.40. Tc00.1047053506713.24. Tc00.1047053511239.60. Tc00.1047053508741.390. Tc00.1047053507275.24. Tc00.1047053506947.50. Tc00.1047053511727.220. Tc00.1047053507083.70. Tc00.1047053507159.30. Tc00.1047053511523.50. Tc00.1047053511517.130. Tc00.1047053507611.270. Tc00.1047053511643.80. Tc00.1047053507165.30. Tc00.1047053478463.10). **obsolete sequences** (21055. 2117. 11281. 21056. 18649. 1404. 1497. 13828. 23196. 16329. 13999). **RHS** (Tc00.1047053506537.10. Tc00.1047053509765.60. Tc00.1047053508877.30. Tc00.1047053506683.210. Tc00.1047053506751.70. Tc00.1047053506129.80. Tc00.1047053506561.20. Tc00.1047053507975.10. Tc00.1047053509559.20. Tc00.1047053413293.30. Tc00.1047053506951.10. Tc00.1047053445777.10. Tc00.1047053506349.83. Tc00.1047053508071.90. Tc00.1047053509259.180. Tc00.1047053504099.70. Tc00.1047053508483.40) |
| Cluster 2 | **ama1 protein. putative** (Tc00.1047053504071.110). **ARP2/3 complex subunit. putative** (Tc00.1047053508737.194). **calpain-like cysteine peptidase. putative** (Tc00.1047053506563.210). **developmentally regulated phosphoprotein. putative** (Tc00.1047053511421.110). EF-hand protein 5. putative (Tc00.1047053506391.30 e Tc00.1047053507483.20). **2.4-dienoyl-coa reductase FADH1. putative** (Tc00.1047053509941.100). **glucose-6-phosphate isomerase. glycosomal. putative** (Tc00.1047053506529.508). **lactoylglutathione lyase-like protein. putative** (Tc00.1047053510659.240). **protein kinase C substrate protein. heavy chain. putative** (Tc00.1047053508215.9). **tryparedoxin peroxidase. putative** (Tc00.1047053508649.5). **hypothetical proteins** (Tc00.1047053509601.140. Tc00.1047053503415.40. Tc00.1047053506825.200. Tc00.1047053503911.30. Tc00.1047053508479.330. Tc00.1047053507491.140. Tc00.1047053506773.104. Tc00.1047053511391.110. Tc00.1047053509393.10. Tc00.1047053509001.30. Tc00.1047053510381.40. Tc00.1047053511421.90. Tc00.1047053506831.63. Tc00.1047053503925.6. Tc00.1047053508153.364. Tc00.1047053506739.99. Tc00.1047053503975.100. Tc00.1047053508707.300. Tc00.1047053504005.54. Tc00.1047053511755.19. Tc00.1047053508059.50. Tc00.1047053504037.10). **obsolete sequence** (19423). **RHS** (Tc00.1047053505915.10. Tc00.1047053507427.30) |
| Cluster 3 | **Alcohol dehydrogenase. putative** (Tc00.1047053506357.50). **aspartate carbamoyltransferase. putative** (Tc00.1047053508375.30). **aspartyl aminopeptidase. putative** (Tc00.1047053508183.4). **co-chaperone GrpE. putative** (Tc00.1047053507929.20). **lactoylglutathione lyase-like protein. putative** (Tc00.1047053510743.70). **tyrosyl-DNA phosphodiesterase. putative** (Tc00.1047053506619.40). **vacuolar sorting protein. putative** (Tc00.1047053508479.290)**. hypothetical proteins** (Tc00.1047053504227.10. Tc00.1047053511467.60. Tc00.1047053503839.19. Tc00.1047053503419.54. Tc00.1047053506789.270. Tc00.1047053507875.30. Tc00.1047053508909.160. Tc00.1047053503841.20. Tc00.1047053503395.20. Tc00.1047053508879.10. Tc00.1047053509245.29. Tc00.1047053506925.310). **obsolete sequences** (18850. 13664. 21269)**. RHS** (Tc00.1047053504285.10. Tc00.1047053507777.30) |
| Cluster 4 | **Glucose-6-phosphate isomerase. glycosomal. putative** (Tc00.1047053506529.508). **lathosterol oxidase. putative** (Tc00.1047053473111.10). **tubulin binding cofactor A-like protein. putative** (Tc00.1047053509069.30). **hypothetical proteins** (Tc00.1047053510535.10. Tc00.1047053509029.20. Tc00.1047053507083.10. Tc00.1047053506829.90. Tc00.1047053504433.20). **obsolete sequences** (11747. 6364) |
| Cluster 5 | **Hypothetical proteins** (Tc00.1047053508319.10. Tc00.1047053509073.60). **obsolete sequence** (5884) |
| Cluster 6 | **Hypothetical proteins** (Tc00.1047053510835.20. Tc00.1047053510173.120. Tc00.1047053511201.40. Tc00.1047053509627.10. Tc00.1047053511671.140). **obsolete sequences** (9204. 14862. 14569) |
| Cluster 7 | **ATP-binding cassette transporter ABCA1. putative** (Tc00.1047053504149.20). **N-acetylglucosamine-6-phosphate deacetylase-like protein. putative** (Tc00.1047053506341.10). **trypanothione/tryparedoxin dependent peroxidase 2. putative** (Tc00.1047053503899.119). **hypothetical protein** (Tc00.1047053506195.290). **obsolete sequence** (6361. 26458) |
| Cluster 8 | **flagellar calcium-binding protein. putative** (Tc00.1047053507891.47). **hypothetical proteins** (Tc00.1047053509141.40. Tc00.1047053506885.30. Tc00.1047053508257.180) |
| Cluster 9 | **3-oxo-5-alpha-steroid 4-dehydrogenase. putative** (Tc00.1047053504427.70). **eukaryotic translation initiation factor 3 subunit 7-like protein. putative** (Tc00.1047053506943.160). **GPR1/FUN34/yaaH family. putative** (Tc00.1047053508179.70). **kinetoplast DNA-associated protein. putative** (Tc00.1047053509793.10). **membrane transporter protein. putative** (Tc00.1047053510667.14). **nucleolar protein. putative** (Tc00.1047053511573.58). **phosphatidic acid phosphatase protein. putative** (Tc00.1047053511355.30). **pumilio/PUF RNA binding protein 7. putative** (Tc00.1047053511715.100). **UDP-Gal or UDP-GlcNAc-dependent glycosyltransferase. putative** (Tc00.1047053503487.50). **hypothetical proteins** (Tc00.1047053510543.80. Tc00.1047053509755.89. Tc00.1047053507053.140. Tc00.1047053508307.90. Tc00.1047053503809.120. Tc00.1047053510857.30) |
| Cluster 10 | **COP-coated vesicle membrane protein erv25 precursor. putative** (Tc00.1047053510187.270). **elongation initiation factor 2 alpha subunit. putative** (Tc00.1047053508153.730). **eukaryotic translation initiation factor 1A. putative** (Tc00.1047053463269.10). **mevalonate kinase. putative (2 copies) (Tc00.1047053436521.9). mitochondrial RNA editing ligase 1. putative** (Tc00.1047053510155.20). **nuclear transport factor 2 protein(NFT2). putative** (Tc00.1047053508173.180). **nuclear transcription factor. putative** (Tc00.1047053510645.20). **trypanothione reductase. putative** (Tc00.1047053503555.30). **hypothetical proteins** (Tc00.1047053507943.40. Tc00.1047053507053.180. Tc00.1047053508173.264. Tc00.1047053509267.40. Tc00.1047053503687.30. Tc00.1047053503897.120. Tc00.1047053509769.60. Tc00.1047053503703.30. Tc00.1047053511071.50). **obsolete sequences** (23161. 5692. 8330) |
| Cluster 11 | **26S proteasome regulatory non-ATPase subunit. putative** (Tc00.1047053504221.20). **40S ribosomal protein S10** (Tc00.1047053506679.150). **40S ribosomal protein S21** (Tc00.1047053510101.430). **40S ribosomal protein** S3 (Tc00.1047053509353.30). **40S ribosomal protein S5** (Tc00.1047053506297.150). **40S ribosomal protein SA** (Tc00.1047053510425.19 e Tc00.1047053503719.20). **60S ribosomal protein L21E** (Tc00.1047053507251.20). **60S ribosomal protein L23** (Tc00.1047053508461.490). **60S ribosomal protein L28** (Tc00.1047053506297.270). **60S ribosomal protein P2** (Tc00.1047053505977.26). **cell differentiation protein. putative** (Tc00.1047053507873.20). **haloacid dehalogenase-like hydrolase. putative** (Tc00.1047053510131.40). **heat shock 70 kDa protein. mitochondrial precursor. putative** (Tc00.1047053511745.10). **chaperonin HSP60. mitochondrial precursor (pseudogene)** (Tc00.1047053510187.420). **D-isomer specific 2-hydroxyacid dehydrogenase-protein. putative** (Tc00.1047053510099.120). **monooxygenase. putative** (Tc00.1047053508173.100). **nucleoside diphosphate kinase. putative** (Tc00.1047053508707.200). **activated protein kinase C receptor. putative** (Tc00.1047053511211.120). **succinyl-CoA synthetase alpha subunit. putative** (Tc00.1047053508479.340). **hypothetical proteins** (Tc00.1047053508719.30. Tc00.1047053504057.80. Tc00.1047053507993.380. Tc00.1047053510099.100. Tc00.1047053447255.10. Tc00.1047053507221.30). **obsolete sequences** (3577. 5161. 4685) |
| Cluster 12 | **2- aminoethylphosphonate:pyruvateaminotransferase-like protein. putative** (Tc00.1047053509693.100). **aminopeptidase P. putative** (Tc00.1047053510655.120). **alpha-tubulin. putative** (Tc00.1047053411235.9). **beta-tubulin. putative** (Tc00.1047053506563.40). **carboxypeptidase. putative** (Tc00.1047053504153.160). **chaperonin. putative** (Tc00.1047053506247.50). **cysteine peptidase. putative** (Tc00.1047053506529.550 e Tc00.1047053508317.10). **cytochrome c1. heme protein. mitochondrial precursor. putative** (Tc00.1047053511391.160). **elongation factor 1-alpha (EF-1-alpha). putative** (Tc00.1047053510119.9). **eukaryotic translation initiation factor 6 (eIF-6). putative** (Tc00.1047053506679.70). **farnesyl pyrophosphate synthase. putative** (Tc00.1047053511823.70). **fatty acid desaturase. putative** (Tc00.1047053511075.9). **fatty acid enlongase. putative** (Tc00.1047053506661.30). **glycosomal phosphoenolpyruvate carboxykinase. putative** (Tc00.1047053507547.90). **hexose transporter. putative** (Tc00.1047053511041.40). **chaperonin HSP60. mitochondrial precursor** (Tc00.1047053507641.280). **NADH-cytochrome B5 reductase. putative** (Tc00.1047053511817.40). **nucleoside transporter-like. putative** (Tc00.1047053506773.50). **pyruvate dehydrogenase E1 beta subunit. putative** (Tc00.1047053510091.80). **prostaglandin F2alpha synthase** (Tc00.1047053507617.9 e Tc00.1047053508461.80). **nucleolar RNA-binding protein. putative** (Tc00.1047053510859.17). **GTP-binding nuclear protein rtb2. putative** (Tc00.1047053503539.30). **25 kDa translation elongation factor 1-beta** (Tc00.1047053507671.30). **thermostable carboxypeptidase 1. putative** (Tc00.1047053504045.60). **thymidine kinase. putative** (Tc00.1047053506855.260). **tryptophanyl-tRNA synthetase (pseudogene)** (Tc00.1047053508421.30). **protein tyrosine phosphatase. putative** (Tc00.1047053511635.40). **V-type ATPase. A subunit. putative** (Tc00.1047053503929.10). **Zn-finger protein. putative** (Tc00.1047053509669.40). **hypothetical proteins** (Tc00.1047053507747.94. Tc00.1047053511517.120. Tc00.1047053507509.40. Tc00.1047053506219.40). **obsolete sequences** (23089. 4010. 24338. 23282. 6794. 6316. 22884. 21242) |
