# Supplementary material for: Trypanosoma cruzi Gene Expression in Response to Gamma Radiation
Source: PLoS One. 2012 Jan 11;7(1):e29596. doi: 10.1371/journal.pone.0029596 (PMC3256153; doi:10.1371/journal.pone.0029596)
Supplement: Table S5 — Fold-change values of DNA repair genes. (DOC) [file pone.0029596.s008.doc]

| Repair pathway and gene name | ID | i.a.i | 4hs | 24hs | 48hs | 96hs | |
| --- | --- | --- | --- | --- | --- | --- | --- |
| **Direct repair** | | | | | | | |
| O-6 methyl-guanine alkyl transferase. putative (MGMT) | Tc00.1047053508347.20 | -1.02 | -1.00 | -1.03 | -1.00 | -1.00 | |
| **Oxidative repair** | | | | | | |  |
| alkylated DNA repair protein. putative (alkB) | Tc00.1047053510687.140 | -1.04 | -1.10 | -1.13 | -1.03 | -1.05 | |
| **Base excision repair - DNA glycosylase** | | | | | | | |
| MutY/Nth/A-G glycosylase | Tc00.1047053511803.20 | -1.10 | 1.15 | 1.12 | 1.03 | 1.01 | |
| uracil-DNA glycosylase | Tc00.1047053511277.330 | -1.01 | 1.01 | -1.05 | -1.00 | -1.10 | |
| **Base excision repair - DNA polymerase** | | | | | | |  |
| mitochondrial DNA polymerase beta. putative | Tc00.1047053503955.20 | -1.07 | -1.08 | 1.07 | -1.04 | -1.09 | |
| **Nucleotide excision repair –Open complex** | | | | | | | |
| TFIIH-XPD/RAD3 | Tc00.1047053511075.30 | -1.12 | -1.11 | 1.09 | 1.08 | 1.04 | |
| **Homologous recombination – Initiation** | | | | | | | |
| meiosis recombination protein SPO11. putative | Tc00.1047053509537.60 | 1.10 | 1.01 | 1.05 | 1.18 | 1.23 | |
| **Homologous recombination - Strand exchange** | | | | | | | |
| meiotic recombination protein DMC1. putative | Tc00.1047053506885.310 | -1.00 | 1.21 | -1.00 | 1.14 | 1.10 | |
| DNA repair protein RAD51. putative | Tc00.1047053503801.30 | -1.27 | 1.06 | -1.10 | 1.14 | 1.03 | |
| DNA repair protein. putative (RAD51-4) | Tc00.1047053503613.30 | 1.01 | -1.00 | -1.07 | -1.04 | -1.09 | |
| **Homologous recombination - Meiosis-specific recombination enzymes** | | | | | | | |
| meiotic recombination protein DMC1. putative | Tc00.1047053506885.310 | -1.00 | 1.21 | -1.00 | 1.14 | 1.10 | |
| meiosis recombination protein SPO11. putative | Tc00.1047053509537.60 | 1.10 | 1.01 | -1.05 | -1.18 | -1.23 | |

i.a.i = immediately after irradiation. DNA repair genes with no fold-change values: **Direct repair:** DNA photolyase. putative (Tc00.1047053509027.10); **Base excision repair - DNA glycosylase:** 8-oxoguanine DNA glycosylase. putative (Tc00.1047053506559.170). MutY/Nth/A-G glycosylase (Tc00.1047053504005.10); **Base excision repair - Flap endonuclease:** flap endonuclease-1 (FEN-1). putative (Tc00.1047053511867.110); **Base excision repair - Apurinic/apyrimidinic endonuclease:** APE-1/exonucleaseIII (Tc00.1047053507083.30). APE-2/APN2/exonucleaseIII (Tc00.1047053504827.50); **Base excision repair - DNA polymerase:** mitochondrial DNA polymerase beta-PAK. putative (Tc00.1047053507063.100); **Nucleotide excision repair - Recognition. global genome repair:** RAD16 (Tc00.1047053506925.140). UV excision repair RAD23-like protein. putative (Tc00.1047053506833.60); **Nucleotide excision repair – Recognition. transcription coupled repair**: CSB/RAD26 (Tc00.1047053508675.20); **Nucleotide excision repair – Open complex:** Replication factorA. RFA1 (Tc00.1047053510901.60). Replication factorA. RFA2 (Tc00.1047053510821.50). TFIIH-SSL1 (Tc00.1047053511907.300). TFIIH-TFB2 (Tc00.1047053506009.80). TFIIH-XPB/RAD25 (Tc00.1047053510149.50). TFIIH-XPB/RAD25 (Tc00.1047053511527.20). XPG/RAD2 (Tc00.1047053510667.40); **Nucleotide excision repair – Incision:** RAD1/XPF (Tc00.1047053509779.10). RAD10/ERCC1 (Tc00.1047053510165.20). XPG/RAD2 (Tc00.1047053510667.40); **Mismatch repair:** MLH1(Tc00.1047053504035.140). MSH2 (Tc00.1047053509643.80). MSH3 (Tc00.1047053508277.180). MSH4 (Tc00.1047053507233.110). MSH5 (Tc00.1047053509617.30). MSH6 (Tc00.1047053510187.430). PMS1 (Tc00.1047053510761.10); **Homologous recombination – Initiation:** MRE11 (Tc00.1047053509099.70). NBS1 (Tc00.1047053506743.180). RAD50 (Tc00.1047053508817.70). RECQ (Tc00.1047053506941.90). RECQ (Tc00.1047053507989.40). SPO11 (Tc00.1047053503619.10); **Homologous recombination – Strand Exchange:** RAD51-3 (Tc00.1047053504153.220). RAD51-5 (Tc00.1047053511837.50). RAD51-6 (Tc00.1047053508075.20). RAD54 (Tc00.1047053505183.40). Replication factorA. RFA1 (Tc00.1047053510901.60). Replication factorA. RFA2 (Tc00.1047053510821.50); **Homologous recombination – Resolution:** MUS81 (Tc00.1047053510065.20); **Homologous recombination – Meiosis-specific recombination enzymes:** MSH4 (Tc00.1047053507233.110). MSH5 (Tc00.1047053509617.30). SPO11(Tc00.1047053503619.10); **0Homologous recombination – Topoisomerases:** Topoisomerase IIA (Tc00.1047053508277.370). Topoisomerase IIA (Tc00.1047053509203.70). DNA polymerase Kappa (Tc00.1047053510131.70). DNA polymerase Zeta. catalytic subunit (Tc00.1047053509769.130). Rev1 (Tc00.1047053508625.90); **Other repair genes:** PARP (Tc00.1047053510173.90); **Non-homologous end-joining:** KU70 (Tc00.1047053503643.10). KU80 (Tc00.1047053511491.50).
